# Central control of dynamic gene circuits governs T cell rest and activation

**DOI:** 10.1038/s41586-024-08314-y

**Published:** 2024-12-11

**Authors:** Maya M. Arce, Jennifer M. Umhoefer, Nadia Arang, Sivakanthan Kasinathan, Jacob W. Freimer, Zachary Steinhart, Haolin Shen, Minh T. N. Pham, Mineto Ota, Anika Wadhera, Rama Dajani, Dmytro Dorovskyi, Yan Yi Chen, Qi Liu, Yuan Zhou, Danielle L. Swaney, Kirsten Obernier, Brian R. Shy, Julia Carnevale, Ansuman T. Satpathy, Nevan J. Krogan, Jonathan K. Pritchard, Alexander Marson

**Affiliations:** 1 Gladstone-UCSF Institute of Genomic Immunology, San Francisco, CA USA; 2 Department of Medicine, University of California, San Francisco, CA USA; 3 Biomedical Sciences graduate program, University of California, San Francisco, CA USA; 4 Quantitative Biosciences Institute (QBI), University of California, San Francisco, CA USA; 5 Division of Allergy, Immunology, and Rheumatology, Department of Pediatrics, Stanford University School of Medicine, Stanford, CA USA; 6 Department of Genetics, Stanford University, Stanford, CA USA; 7 Department of Pathology, Stanford University School of Medicine, Stanford, CA USA; 8 Gladstone Institute of Data Science and Biotechnology, San Francisco, CA USA; 9 Department of Cellular and Molecular Pharmacology, University of California, San Francisco, CA USA; 10 Department of Laboratory Medicine, University of California, San Francisco, CA USA; 11 UCSF Helen Diller Family Comprehensive Cancer Center, University of California, San Francisco, CA USA; 12 Parker Institute for Cancer Immunotherapy, San Francisco, CA USA; 13 Department of Bioengineering and Therapeutic Sciences, University of California, San Francisco, CA USA; 14 Department of Biology, Stanford University, Stanford, CA USA; 15 Innovative Genomics Institute, University of California-Berkeley, Berkeley, CA USA; 16 Department of Microbiology and Immunology, University of California, San Francisco, CA USA; 17 Institute for Human Genetics, University of California, San Francisco, CA USA

**Keywords:** Gene regulation in immune cells, Gene regulatory networks

## Abstract

The ability of cells to maintain distinct identities and respond to transient environmental signals requires tightly controlled regulation of gene networks^1–3^. These dynamic regulatory circuits that respond to extracellular cues in primary human cells remain poorly defined. The need for context-dependent regulation is prominent in T cells, where distinct lineages must respond to diverse signals to mount effective immune responses and maintain homeostasis^4–8^. Here we performed CRISPR screens in multiple primary human CD4^+^ T cell contexts to identify regulators that control expression of IL-2Rα, a canonical marker of T cell activation transiently expressed by pro-inflammatory effector T cells and constitutively expressed by anti-inflammatory regulatory T cells where it is required for fitness^9–11^. Approximately 90% of identified regulators of IL-2Rα had effects that varied across cell types and/or stimulation states, including a subset that even had opposite effects across conditions. Using single-cell transcriptomics after pooled perturbation of context-specific screen hits, we characterized specific factors as regulators of overall rest or activation and constructed state-specific regulatory networks. MED12 — a component of the Mediator complex — serves as a dynamic orchestrator of key regulators, controlling expression of distinct sets of regulators in different T cell contexts. Immunoprecipitation–mass spectrometry revealed that MED12 interacts with the histone methylating COMPASS complex. MED12 was required for histone methylation and expression of genes encoding key context-specific regulators, including the rest maintenance factor KLF2 and the versatile regulator MYC. CRISPR ablation of MED12 blunted the cell-state transitions between rest and activation and protected from activation-induced cell death. Overall, this work leverages CRISPR screens performed across conditions to define dynamic gene circuits required to establish resting and activated T cell states.

## Abstract (web summary)

Resting and activated T cell states are established by context-specific regulators and dynamic gene circuits.

## Main

Each cell type expresses a distinctive set of genes to maintain its identity and respond to external cues. Context-specific networks of *trans*-regulatory proteins are required to coordinate these gene expression programs but are not fully mapped in human cells^1,12,13^. The intricacies of conditional gene regulation are exemplified within the human immune system, where diverse cell types must specialize as well as sense and respond dynamically to stimuli to maintain homeostasis^5,7^. Cell-type-specific and context-specific expression of receptors and other key molecules enable coordinated immune responses and have been targeted in immune-modulating therapies^14–16^. However, the *trans*-regulatory mechanisms that allow for conditional expression of the genes encoding these proteins remain poorly understood. Deciphering these systems will advance our understanding of nuanced gene regulation required for human health and improve our ability to modulate the immune system with effective immunotherapies.

Within the CD4^+^ T cell compartment, regulatory T (T_reg_) cells and effector T (T_eff_) cells functionally oppose each other, serving immunosuppressive and immunostimulating roles, respectively. However, their relatively late-stage differentiation results in a high degree of similarity at the gene expression level between the two cell types^4,5,8^. Both possess the ability to respond to a set of shared environmental signals, albeit with key differences. The cytokine IL-2 drives cellular fitness of T_reg_ cells and activated T_eff_ cells, and competition for this signal can shape immune responses in health and disease^10,17^. The IL-2 receptor high-affinity subunit IL-2Rα (also known as CD25) enhances receptor affinity for IL-2 and is carefully regulated to control sensitivity to the cytokine^11,15^. T_reg_ cells constitutively express high levels of IL-2Rα at rest and mildly increase expression of the receptor upon activation, whereas T_eff_ cells express low levels of IL-2Rα at rest but transiently upregulate the receptor for days following TCR stimulation^9^. Numerous therapeutic strategies have been used to improve the cellular specificity and longevity of IL-2 signalling, some of which utilize the distinct expression patterns of IL-2Rα across subsets to promote efficacy and prevent adverse events^15,18^. *IL2RA* represents a clinically relevant gene to study for mechanistic insights into cell-type-specific and stimulation-specific gene regulation.

## Context-specific IL-2Rα regulator screens

**Fig. 1:**
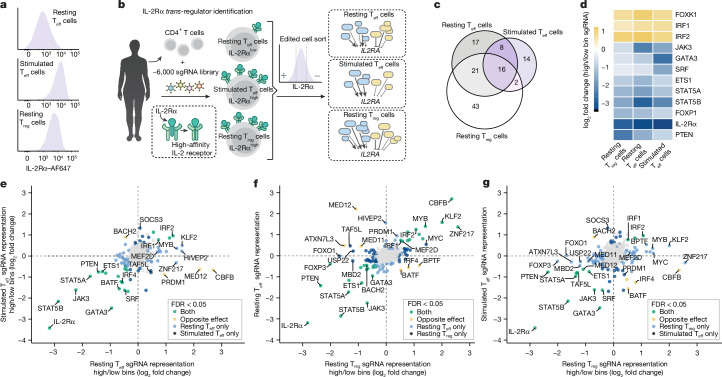
Identification of context-dependent regulators of IL-2Rα expression. **a**, IL-2Rα surface expression levels by flow cytometry. **b**, Schematic of the context-specific *trans*-regulatory CRISPR screens. Schematic includes content from S. Pyle and BioRender (https://biorender.com). **c**, Venn diagram of regulators identified across screen conditions. **d**, Consistent regulators of IL-2Rα identified as significant in the same direction across all three screens (FDR < 0.05; *n* = 2 donors for the T_reg_ screen, *n* = 3 donors for the resting T_eff_ screen and *n* = 3 donors for the stimulated T_eff_ screen). **e**–**g**, Comparisons of IL-2Rα screen results (resting versus stimulated T_eff_ IL-2Rα screens (**e**), resting T_reg_ versus resting T_eff_ IL-2Rα screens (**f**) and resting T_reg_ versus stimulated T_eff_ IL-2Rα screens (**g**)) coloured by significance and direction of effect in both screens (significant denotes FDR < 0.05; *n* = 2 donors for the T_reg_ screen, *n* = 3 donors for the resting T_eff_ screen and *n* = 3 donors for the stimulated T_eff_ screen).

**Extended Data Fig. 1:**
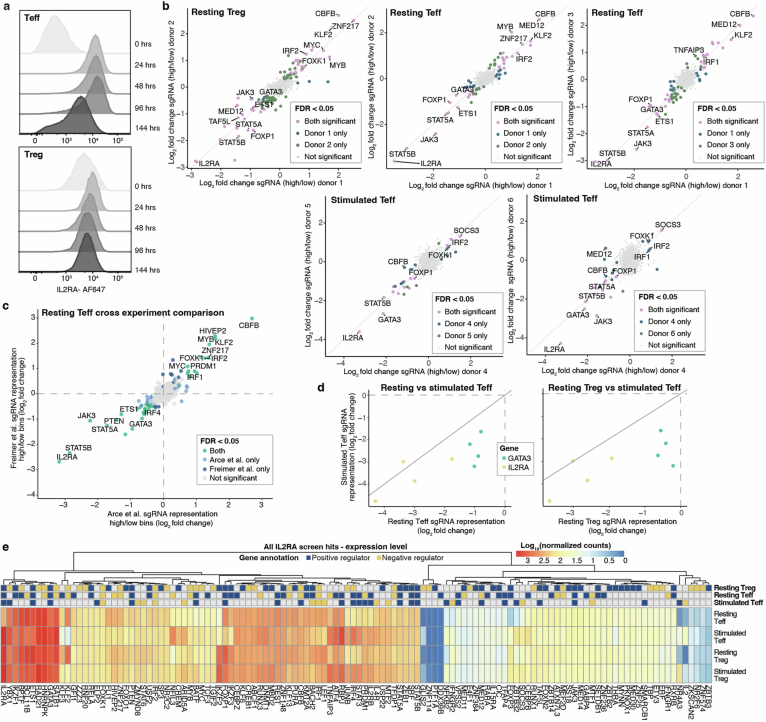
Pooled KO screens across cell states and lineages reveal context-specific regulators. **a**. Kinetics of IL-2Rα expression in Teffs and Tregs following restimulation. Representative histograms of IL-2Rα expression assessed via flow cytometry adjusted to the mode of each sample. **b**. Donor-to-donor correlations for all screening conditions (FDR < 0.05; Treg screen: n = 2, Teff screen: n = 3, Stimulated Teff screen: n = 3 donors). **c**. Comparison of resting IL-2Rα screen results between Freimer et al. and new screen data. Non-significant genes are shown in grey and significant hits (FDR < 0.05) colored by direction of effect in both screens. **d**. Comparison of IL-2Rα screen results for IL-2Rα KO and GATA3 KO sgRNAs. Each dot represents an individual sgRNA average effect for the respective gene KO (Treg screen: n = 2, Teff screen: n = 3, Stimulated Teff screen: n = 3 donors). **e**. Heatmap showing expression of genes encoding screen hits. Transcript levels were assessed in *AAVS1* KO control cells by bulk RNAseq. The color bar represents the log10(mean normalized counts) for the gene expression level of each IL-2Rα regulator across each cell type and stimulation condition (n = 3 donors). The annotation bars on the top illustrate the direction of effect for each regulator in the three IL-2Rα screens (colored boxes = FDR < 0.05, navy = positive regulator of IL-2Rα and gold = negative regulator of IL-2Rα). Source Data

We applied pooled CRISPR knockout screens to identify upstream *trans*-regulators of IL-2Rα across cell-type and stimulation conditions. We utilized a library of 6,000 single guide RNAs (sgRNAs) to target *trans*-factor genes expressed in T cells (approximately 1,350 transcription factors and chromatin modifiers) as well as select immune regulators and control genes^19,20^. We isolated, edited and expanded primary human T_reg_ cells (CD4^+^IL-2Rα^high^CD127^low^) and T_eff_ cells (CD4^+^IL-2Rα^low^). We then screened for regulators of IL-2Rα in resting T_eff_ cells (IL-2Rα^low^) and resting T_reg_ cells (IL-2Rα^high^) 10 days after initial stimulation, as well as restimulated T_eff_ cells (IL-2Rα^high^; 72 h post-stimulation; Fig. 1a,b and Extended Data Fig. 1a). Screens were performed at high coverage (700–1,000× cells per sgRNA per donor) and had similar positive control sgRNA effect sizes across conditions, as well as high donor-to-donor correlations and the resting T_eff_ screen replicated published results^19^ (Extended Data Fig. 1b–d).

The screens collectively detected over 100 *trans*-regulators (FDR < 0.05; Fig. 1c and Supplementary Table 1) whose perturbation altered IL-2Rα surface expression in at least one context. Only 16 regulators were hits in all three screens, 75% of which shared the same direction of effect across conditions (Fig. 1d). These 12 ‘consistent regulators’ of IL-2Rα included members of the JAK–STAT pathway. Among the consistent regulators, the effect sizes of several *trans*-regulators varied greatly between conditions. GATA3, for example, was a particularly potent positive regulator of IL-2Rα in stimulated T_eff_ cells, with a median log_2_ fold change in sgRNA enrichment in the IL-2Rα low/high bin of 2.47 compared with 0.95 and 0.49 in resting T_eff_ cells and resting T_reg_ cells, respectively (Fig. 1d and Extended Data Fig. 1d). The majority of identified IL-2Rα regulators were significant in only one or two conditions, demonstrating cell-type-specific or stimulation-specific effects (Fig. 1c), although most regulators were expressed (based on bulk RNA sequencing (RNA-seq)) across conditions (Extended Data Fig. 1e). We compared the direction and magnitude of effect of the perturbations across the three screens to categorize context-dependent regulators of IL-2Rα. Of note, few strong negative regulators were identified in stimulated T_eff_ cells compared with resting T_eff_ cells. For example, KLF2, MYB and ZNF217 were only identified as significant negative regulators in the resting state (Fig. 1e). These data highlight broad differences in the network upstream of IL-2Rα between activation states, including fewer negative-regulatory forces following stimulation.

**Extended Data Fig. 2:**
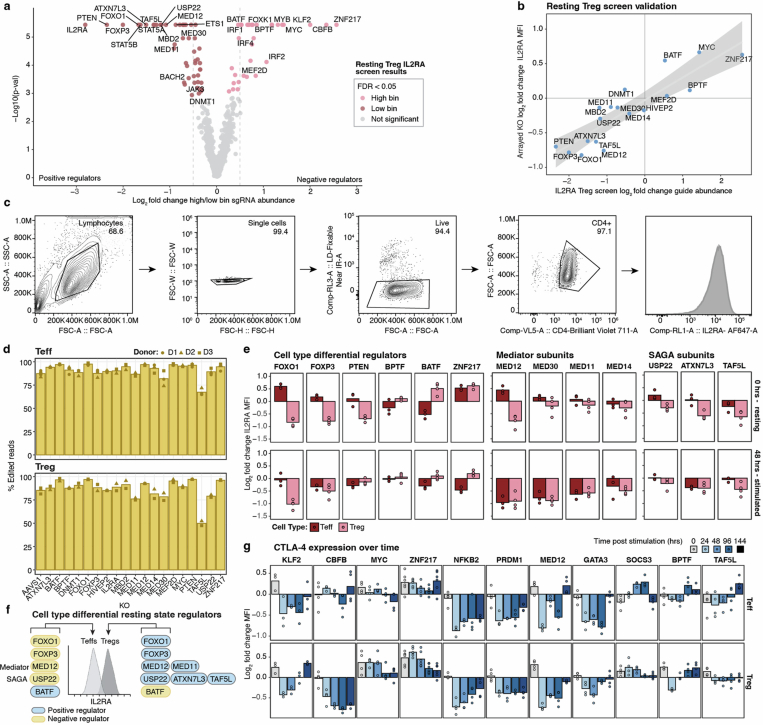
Cell type- and stimulation-specific regulators of IL-2Rα control dynamic gene expression. **a**. Volcano plot of screen results for regulators of IL-2Rα in resting primary human Tregs. Significant hits (FDR < 0.05, n = 2 donors) colored by direction of effect. **b**. Validation of select Treg screen hits using arrayed KO and flow cytometry. IL-2Rα expression displayed as the mean log_2_ fold change median fluorescent intensity (MFI) of the perturbed samples compared to control *AAVS1* KO control samples vs. the log_2_ fold change IL-2Rα Treg screen effect (flow cytometry n = 4 donors; Treg screen n = 2 donors). **c**. Flow cytometry gating strategy for IL-2Rα expression in arrayed assays displayed as contour plots with outliers. **d**. Amplicon-seq genotyping of arrayed validation KOs to confirm editing. The mean percent modified (edited) reads as quantified by NGS is shown on the y axis for each of the targeted genes (Teff genotyping n = 3, Treg genotyping n = 2 donors). **e**. Select regulators of IL-2Rα demonstrate cell type-specific effects. Regulators from arrayed KO in b selected for visualization based on apparent cell type differential effect and membership to Mediator or SAGA. IL-2Rα surface expression displayed as the log_2_ fold change median fluorescent intensity (MFI) of the perturbed samples compared to control *AAVS1* KO sample from the same donor (Teffs: n = 3 donors, Tregs: n = 4 donors). **f**. Schematic of IL-2Rα screen hits with cell type-differential regulatory roles. The schematic was created using BioRender (https://biorender.com). **g**. Intracellular CTLA-4 expression is affected by perturbation of stimulation-responsive regulators of IL-2Rα. Data displayed as described in e., but for CTLA-4 expression. (n = 2 donors x 2 sgRNAs per KO except KLF2 KO where n = 3 and ZNF217 KO where n = 6) ZNF217 KO and *AAVS1* KO data of CTLA-4 for time 0 Tregs was published in Mowery et al.^87^. Gating strategy is as displayed in c. Source Data

Although both T_reg_ cells and activated T_eff_ cells express high levels of IL-2Rα, there were fewer shared IL-2Rα regulators between the resting T_reg_ and stimulated T_eff_ screens than the resting T_reg_ and resting T_eff_ screens, indicating that T_reg_ cells and stimulated T_eff_ cells rely on different systems to achieve high expression (Fig. 1c–g). Overall, the screen performed in T_reg_ cells yielded a particularly large number of significant hits, including both positive and negative regulators specific to the condition, such as FOXO1, USP22 (ref. ^21^) and MYC (Extended Data Fig. 2a). IL-2Rα is required for the fitness of T_reg_ cells^11^, and the large network of positive and negative regulators probably acts as a buffering system to maintain relatively consistent expression.

A few regulators exerted effects in opposing directions across conditions. Of note, MED12, CBFB and PRDM1 were identified as positive regulators of IL-2Rα in stimulated T_eff_ cells but strong negative regulators in resting T_eff_ cells (Fig. 1e). MED12 and, to a lesser extent, MED11 — components of Mediator of RNA polymerase II (Mediator) — were both identified as positive regulators of IL-2Rα in resting T_reg_ cells but negative regulators of IL-2Rα in resting T_eff_ cells. These strong ‘differential’ context-dependent effects were particularly striking for components of a complex with general roles in transcription. In addition, BATF and IRF4, which co-bind genomic sites in T cells^22^, were identified as differential regulators with negative effects on IL-2Rα levels in resting T_reg_ cells and positive effects on IL-2Rα expression in both resting and stimulated T_eff_ cells (Fig. 1f,g). Of note, BATF has been highlighted as a key regulator of T_reg_ tissue homing and stability in vivo^23,24^. Our characterization of BATF and IRF4 as negative regulators of IL-2Rα in ex vivo human T_reg_ cells suggests a nuanced role with potential differences across species or contexts. Comprehensively, the screening approach led to the identification of cell-type-specific and stimulation-specific regulators upstream of IL-2Rα, as well as the unexpected class of regulators that promote and repress expression of IL-2Rα in distinct contexts.

## Dynamic regulation of IL-2Rα

**Fig. 2:**
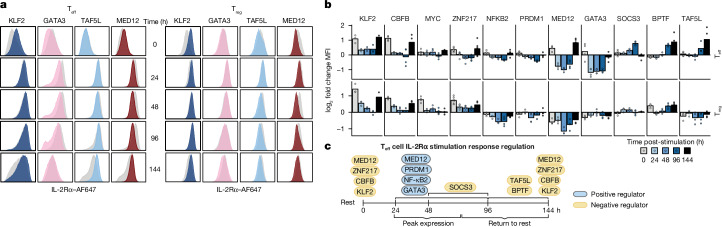
Temporal regulation of IL-2Rα following stimulation by distinct factors. **a**, Representative flow cytometry histograms of IL-2Rα expression after arrayed knockout. *AAVS1* safe harbour control knockout results are shown in grey, and the *y* axis is normalized to the mode. Timepoints represent time after restimulation starting with 0 h (no restimulation). **b**, Quantification of the knockout effect on IL-2Rα expression across stimulation timepoints for select regulators. log_2_ Fold change IL-2Rα median fluorescent intensity (MFI) calculated for knockout compared with *AAVS1*-knockout control samples from the same donor. Each point represents a donor and sgRNA combination (*n* = 2 donors × 2 sgRNAs per knockout, except KLF2 knockout where *n* = 3 and ZNF217 knockout where *n* = 6). **c**, Schematic of select IL-2Rα regulators that enable temporal control of IL-2Rα in T_eff_ cells. The schematic was created using BioRender (https://biorender.com). Source Data

To validate and characterize the function of cell-type-specific hits from our screens, we ablated 18 factors and quantified IL-2Rα protein expression in both T_reg_ cells and T_eff_ cells, prioritizing genes with discordant effects across cell types or T_reg_-specific effects. The arrayed CRISPR knockout results confirmed condition-specific regulatory roles for many factors (Extended Data Fig. 2b–e). Components of Mediator (MED12, MED11 and MED30) and SAGA (TAF5L, USP22 and ATXN7L3), which are both ubiquitous transcriptional coactivator complexes, demonstrated cell-type-specific and stimulation-specific effects. Consistent with screen results, MED12 had the most dynamic role (Extended Data Fig. 2e,f) despite steady expression levels across conditions (Extended Data Fig. 1e). Ablation of MED12 consistently increased the levels of IL-2Rα in resting T_eff_ cells but decreased the levels of IL-2Rα in stimulated T_eff_ cells and in T_reg_ cells (both resting and stimulated). We dissected the kinetics of stimulation-responsive regulation through arrayed knockout with an extended series of collection timepoints. Much like the screen, many perturbations that increased IL-2Rα in resting T_eff_ cells (for example, ZNF217, MED12 and PRDM1) had minimal effects on IL-2Rα expression or even decreased its expression 48–72 h after stimulation (Fig. 2a,b). By contrast, fewer IL-2Rα regulators with distinct stimulation-responsive effects were observed in T_reg_ cells than in T_eff_ cells; no negative regulators of IL-2Rα in resting T_reg_ cells became positive regulators during activation or vice versa. Despite differences in activation responses, both T_reg_ cells and T_eff_ cells appeared reliant on KLF2 and CBFB to repress IL-2Rα at resting timepoints (Fig. 2a,b).

Although our pooled screens captured regulators of maximum and minimum levels of IL-2Rα expression at specific timepoints, the arrayed knockout time course experiments also revealed regulators that govern transitions between states. We identified several factors that enable the transition from activated IL-2Rα levels to rest levels (approximately 96–144 h) with particularly large effects in T_eff_ cells, which undergo the greatest fluctuations in IL-2Rα expression. TAF5L, BPTF and SOCS3 contributed to this reduction of surface IL-2Rα as cells returned to rest (Fig. 2b,c). CTLA-4, another receptor that is transiently induced in stimulated T_eff_ cells and constitutively expressed in T_reg_ cells, exhibited similar patterns of temporal regulation by the perturbed genes, suggesting that the regulators control a broader network of dynamically expressed genes (Extended Data Fig. 2g). In summary, many regulators contribute to activation-associated and rest-associated gene regulation in temporally defined stages, with some regulatory systems specific to each T cell subset.

## MED12 facilitates rest and activation

**Fig. 3:**
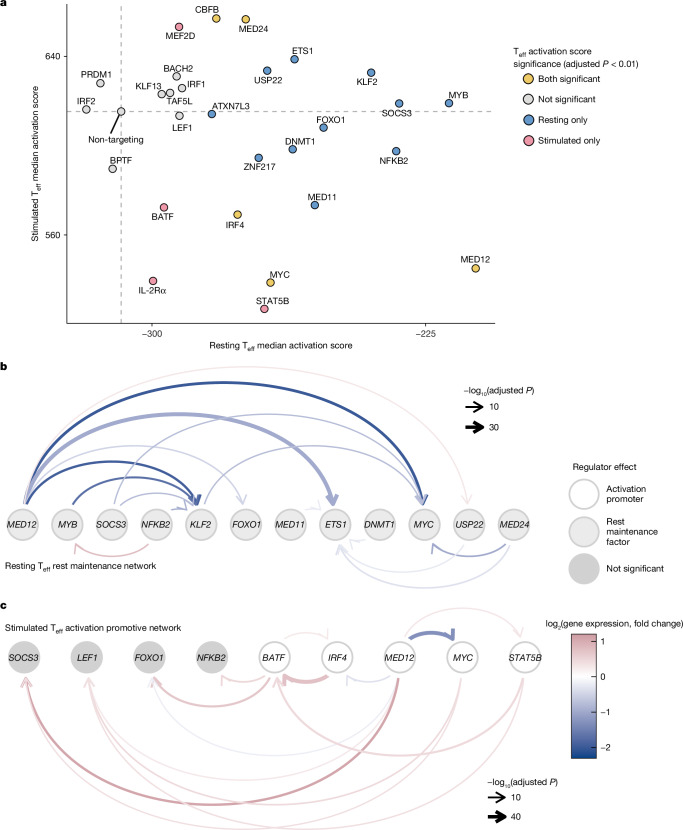
Perturb-seq reveals regulator networks controlling T cell rest and activation. **a**, Activation scores computed for each perturbed gene based on single-cell gene signatures across resting and stimulated states. Each point represents the median activation score of cells targeted for CRISPRi knockdown of the indicated gene. The grey dashed lines indicate the activation scores for non-targeting control cells. The coloured dots indicate perturbation with activation scores significantly different compared with control cells for each condition, determined by a two-sided Wilcoxon rank-sum test with continuity correction (adjusted *P* < 0.01). **b**,**c**, Regulatory network of factors controlling rest (**b**) and activation (**c**). Differentially expressed genes resulting from a perturbation (identified by pseudo-bulking knockdown versus non-targeting cells) are represented as arrows from the perturbed gene (Wald test with Benjamini–Hochberg multiple test correction, adjusted *P* < 0.05 threshold; *n* = 2 donors per target gene). The light grey nodes indicate rest maintenance factors in resting T_eff_ cells. The white nodes indicate activation-promoting factors in stimulated T_eff_ cells. The dark grey nodes indicate regulators without significant effects on activation scores in stimulated T_eff_ cells (categorization from panel **a**). Source Data

**Extended Data Fig. 3:**
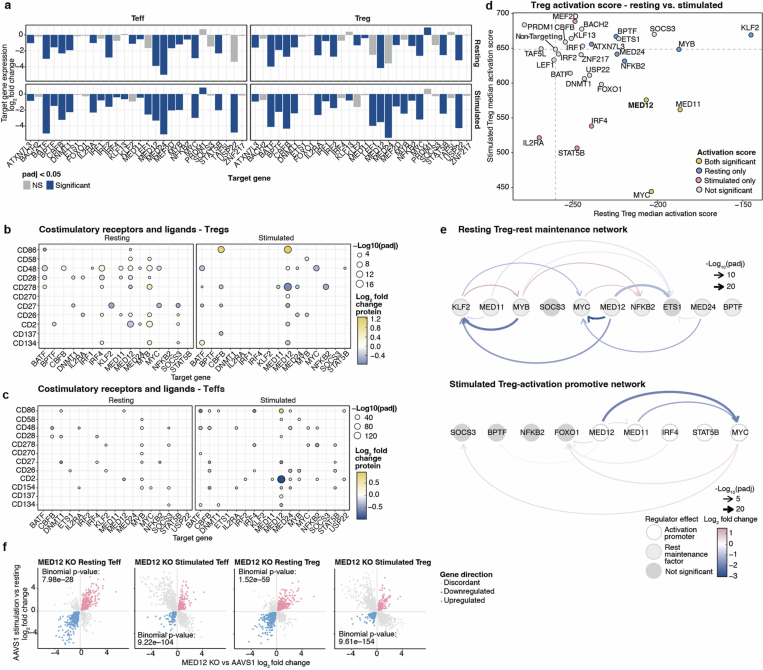
MED12 controls expression of stimulation-responsive genes in Tregs and Teffs. **a**. Differential gene expression results for CRISPRi targeted genes using pseudobulked Perturb-seq counts relative to non-targeting control cells (Wald test with BH multiple test correction, padj <0.05, n = 2 donors). **b-c**. Differentially expressed cell surface proteins from pseudobulked Perturb-CITE-seq samples (Wald test with Benjamini-Hochberg (BH) multiple test correction, padj <0.05, n = 2 donors). **d**. Activation scores for each perturbed gene computed based on single cell gene signatures. Each point represents the median activation score of cells targeted for CRISPRi knock-down of the indicated gene in Tregs. Dashed grey lines indicate the activation score for non-targeting control cells within each respective condition; colored points indicate perturbation with activation scores significantly different than control cells for each condition as determined by a two-sided Wilcoxon rank sum test with continuity correction (padj <0.01). **e**. Regulatory network of factors controlling rest and activation in Tregs. Differentially expressed genes resulting from a perturbation (identified by pseudo-bulking knock-down vs. non-targeting cells) are represented as arrows from the perturbed gene (padj <0.05, n = 2 donors per target gene). Light grey nodes indicate rest maintenance factors in resting Tregs, dark grey nodes indicate regulators without significantly different activation scores in the respective condition, and white nodes indicate activation promoting regulators in stimulated Tregs **f**. Log_2_ fold change of differentially expressed genes in MED12 KO vs *AAVS1* KO bulk RNAseq samples (padj <0.05 as described in 4a) compared to the log_2_ fold change of differentially expressed genes between stimulated and resting control *AAVS1* KO cells (Wald test with Benjamini-Hochberg (BH) multiple test correction, padj <0.05, n = 3 donors). Two-sided binomial test results are displayed comparing the proportion of genes downstream of MED12 that are concordant in direction with stimulation-responsive genes to genes discordant in direction. Source Data

Stimulation-induced expression of IL-2Rα is a canonical marker of T cell activation. We suspected that many of the effects of the regulators were not limited to IL-2Rα and were reflections of altered overall activation states. To characterize such global effects, we performed Perturb-CITE-seq (pooled CRISPR perturbations coupled with single-cell RNA-seq and surface proteomics) in resting and stimulated (48 h post-stimulation) T_reg_ cells and T_eff_ cells. We used CRISPR interference (CRISPRi), to knock down 28 regulators of IL-2Rα, prioritizing *trans*-factors with state-specific effects. We confirmed perturbation efficiency via transcript expression of the targeted regulator and observed significant changes to the transcriptome and key cell-surface receptors (Extended Data Fig. 3a–c). We next assessed resulting changes to the overall T cell activation states based on a global transcriptional signature^25^. Many context-specific regulators of IL-2Rα served as broad modulators of rest or activation, confirming our hypothesis. In resting T_eff_ cells, KLF2, MYB and SOCS3 stood out as strong repressors of activation, whereas STAT5B, MYC, BATF and IRF4 appeared particularly important to promote activation in stimulated cells (Fig. 3a). Most notably, knockdown of MED12 increased the activation scores of resting cells but lowered the activation score of stimulated cells in both cell types (Fig. 3a and Extended Data Fig. 3d). Collectively, these results reveal core regulators of global state-specific gene expression within our screen hits and distinguish MED12 as a dynamic factor governing both rest and activation programs.

Cell-state regulators often operate in hierarchical networks^26^. We constructed state-specific and cell-type-specific gene regulatory network maps to visualize how regulators affect one another (Fig. 3b,c, Extended Data Fig. 3e and Supplementary Table 2). Among rest maintenance factors, there were many positive-regulatory connections converging on *KLF2*, *ETS1* and *MYC*, which were downstream of the largest number of genes (Fig. 3b and Extended Data Fig. 3e). Of note, MED12 strongly promoted expression of these core resting-state maintenance factors. The network structure of regulators controlling gene expression in stimulated T_eff_ cells was distinct from that in resting T_eff_ cells. In stimulated T_eff_ cells, we found few instances of strong positive connections, with the exception of MED12 promoting the expression of *MYC* (Fig. 3c). Instead, MED12, MYC, STAT5B and BATF (all factors that promote activation following stimulation; white, Fig. 3c) were required to repress expression of genes encoding several resting-state maintenance factors that did not affect activation following stimulation, including SOCS3, NF-κB2 and FOXO1 (coloured in dark grey, Fig. 3c). This network structure echoes the general structure observed in our IL-2Rα screens and arrayed assays, in which we found reduced negative-regulatory effects following stimulation relative to the resting state. Even SOCS3, the strongest negative regulator of IL-2Rα identified in the stimulated T_eff_ screen, was more specifically characterized as an early return-to-rest repressor of IL-2Rα (Fig. 2b). Perturb-seq further clarifies that SOCS3 and other rest maintenance or rest-promoting factors are repressed in stimulated cells by activation-promoting factors, allowing the cells to transiently reach an activated state. These results lead to a model in which the resting state is actively reinforced by a self-promoting network of regulators, and the transition to peak activation state requires repression of factors that promote rest. MED12 orchestrates the expression of key regulators of both rest and activation within these networks.

## MED12 controls key regulators of IL-2Rα

**Fig. 4:**
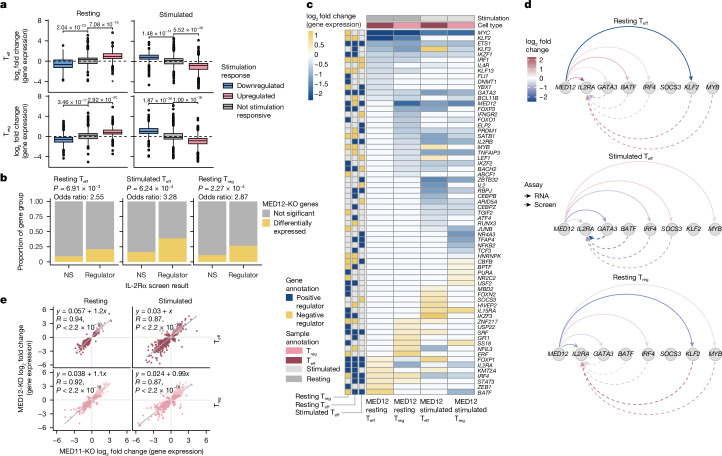
MED12 coordinates expression of IL-2Rα regulators across CD4^+^ T cell conditions. **a**, Genes differentially expressed in MED12-knockout samples compared with control *AAVS1*-knockout samples (Wald test with Benjamini–Hochberg multiple test correction, adjusted *P* < 0.05; *n* = 3 donors per knockout) are grouped according to their stimulation-responsive behaviour in *AAVS1*-knockout control cells. The Bonferroni-adjusted *P* value resulting from a two-tailed *t*-test is displayed comparing each stimulation-responsive group to the non-stimulation-responsive group (Methods). Box plot centre line denotes the median; box limits indicate upper and lower quartiles; and whiskers denote 1.5× interquartile range. **b**, Proportions of IL-2Rα regulators versus non-regulators (NSs) whose expression levels are affected by MED12 knockout (KO). One-sided Fisher’s exact test for regulators of IL-2Rα downstream of MED12 (Methods) was used. **c**, Heatmap of IL-2Rα regulators differentially expressed between MED12-knockout cells and control cells (as described in **a**). Gene annotation boxes represent the result of the IL-2Rα screens (FDR < 0.05; navy denotes a positive regulator of IL-2Rα, and gold represents a negative regulator of IL-2Rα). **d**, Directed network plots depicting select *trans*-regulators downstream of MED12. The solid lines depict effects of MED12-knockout based on significant gene expression changes as described in **a**, and the dashed lines represent effects on IL-2Rα based on the screen results as described in Fig. 1e–g. **e**, Comparison of transcriptional effects of MED12 ablation versus ablation of the core Mediator subunit MED11. Each point represents the effect on genes significantly differentially expressed in both knockouts, as described in **a**. Linear regression equation and Pearson coefficient are provided for each condition. Source Data

To further probe the mechanism of dynamic regulation by MED12, we knocked out the gene and performed bulk RNA-seq. In both T_eff_ and T_reg_ cells, knockout of MED12 caused resting cells to prematurely upregulate or downregulate genes that are normally differentially expressed in response to stimulation (as assessed in *AAVS1*-knockout control cells; Fig. 4a). Conversely, in stimulated cells, we observed dampening of stimulation-induced changes in gene expression in MED12-knockout relative to *AAVS1*-knockout cells. A binomial test also confirmed aberrant expression of stimulation-specific genes in all MED12-knockout conditions (Extended Data Fig. 3f). Together, along with our Perturb-seq activation scoring, these results demonstrate that without MED12, CD4^+^ T cells are unable to reach a full rested state or achieve peak levels of activation and instead exist in an intermediate state.

**Extended Data Fig. 4:**
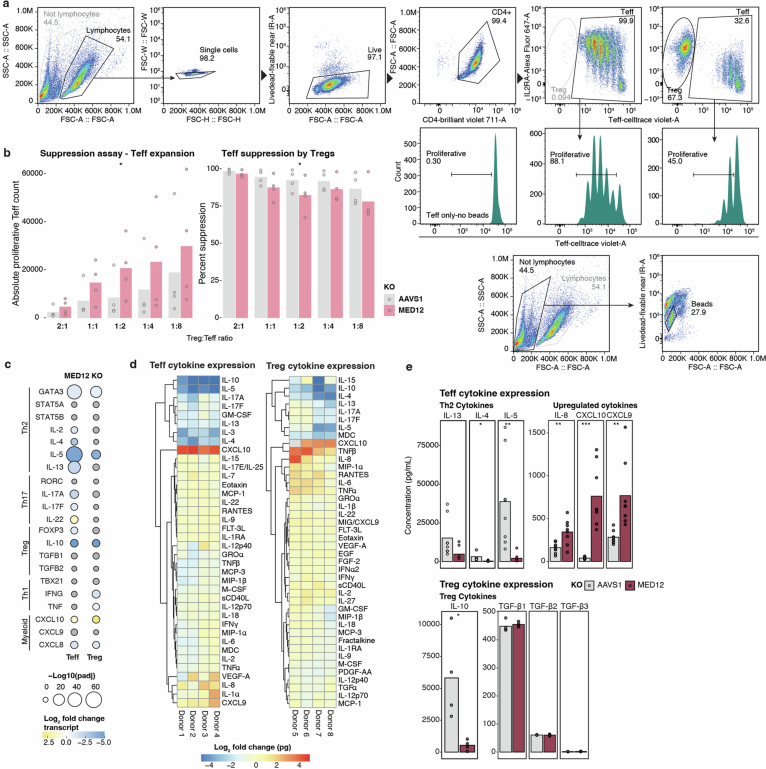
MED12 is required for distinct functional features in specific CD4 + T cell subsets. **a**. Flow gating strategy for suppression assays. **b**. In vitro Treg suppression assays showing absolute proliferative Teff count and percent suppression by Tregs (Methods). Unedited (wild type) CD4+ Teffs were used in the assay with MED12 KO Tregs or *AAVS1* KO control Tregs. Paired two-tailed T test comparing MED12 KO and *AAVS1* KO control samples (n = 4 donors per KO; Teff count: Ratio 2:1 p = 0.11, 1:1 p = 0.067, 1:2 p = 0.023, 1:4 p = 0.12, 1:8 p = 0.15; Suppression: Ratio 2:1 p = 0.067, 1:1 p = 0.061, 1:2 p = 0.045, 1:4 p = 0.24, 1:8 p = 0.14). **c**. Gene expression of selected genes associated with indicated cell identities. Color indicates the log_2_ fold change of differentially expressed genes in bulk RNAseq (Wald test with BH multiple test correction, padj <0.05, n = 3 donors) comparing stimulated MED12 KO to *AAVS1* KO control samples. Data are shown in Teffs (left) and Tregs (right). **d**. Cytokine expression measured by Luminex. Heatmaps represent the log_2_ fold change cytokine concentration in the MED12 KO sample supernatant relative to *AAVS1* KO control supernatant. Teff heatmap values display the average concentration of 2 sgRNAs per gene KO (n = 4 donors per cell type x 2 sgRNA for Teff or 1 sgRNA for Tregs). **e**. Cytokine concentrations as represented in d for select cytokines. Two-tailed T test comparing MED12 KO and *AAVS1* KO samples (paired for Tregs only) (n = 4 donors x 2 sgRNAs per KO (Teff only); Teff cytokines: IL-13: p = 0.054, IL-4: p = 0.025, IL-5: p = 0.0099, IL-8: p = 0.0095, CXCL10 (IP-10): p = 0.00072, CXCL9: p = 0.0096; Treg cytokines: IL-10: p = 0.043, TGF-β1: p = 0.54, TGF-β2: p = 0.84, TGF-β3: p = 0.19). Source Data

Overall, regulators of IL-2Rα identified in our pooled screens were enriched in the differentially expressed genes downstream of MED12 across all conditions and revealed routes of context-specific regulation by MED12 (Fig. 4b and Supplementary Table 3). For example, knockout of MED12 caused increased expression of *IRF4* in resting T_eff_ and T_reg_ cells, but decreased *IRF4* levels in the stimulated cell conditions (Fig. 4c). Ablation of MED12 markedly decreased levels of the positive IL-2Rα regulator *GATA3* in stimulated T_eff_ cells, and decreased levels of the negative IL-2Rα regulator (and rest-maintenance factor) *KLF2* in resting T_eff_ and T_reg_ cells (Fig. 4c,d). Additional experiments revealed functional changes resulting from transcriptional reprogramming of MED12-knockout cells, including reduced suppressive capacity in vitro by T_reg_ cells relative to *AAVS1*-knockout cells, impaired IL-10 secretion by T_reg_ cells, and impaired T helper 2 (T_H_2)-associated cytokine secretion by T_eff_ cells (Extended Data Fig. 4a–e). Collectively, these results reveal that MED12 directs a network composed of cell-type-specific and stimulation-specific regulators to achieve context-dependent expression.

**Extended Data Fig. 5:**
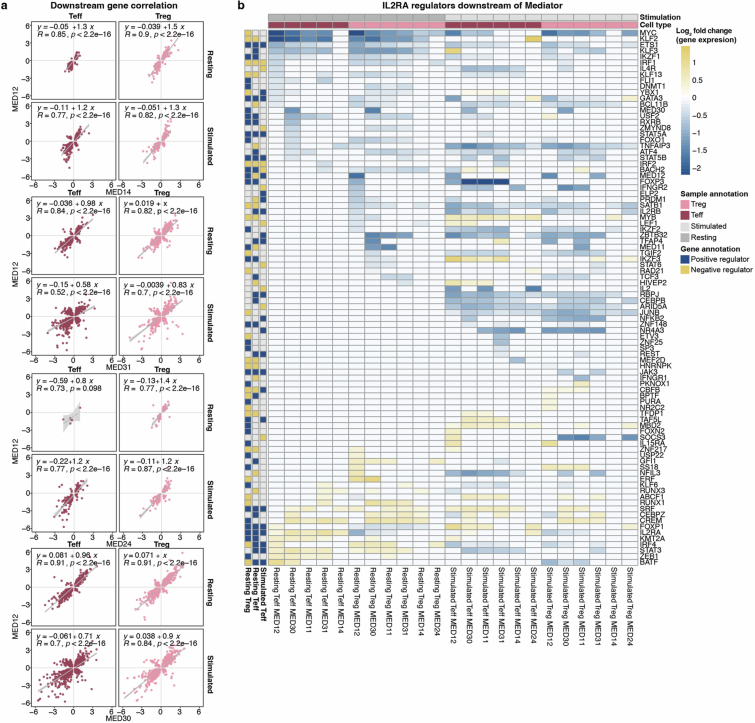
Partially-shared transcriptional effects of MED12 and other Mediator subunits. **a**. Comparison of the transcriptional effects of MED12 KO compared to KO of Mediator subunits MED14 (middle/backbone), MED31 (middle), MED24 (tail), MED30 (tail) as described in Fig. 4e. **b**. Regulators of IL-2Rα in the network downstream of several Mediator complex subunits. Differentially expressed genes as assessed by bulk RNAseq are displayed as the log_2_ fold change gene expression in the subunit KO/*AAVS1* KO samples for the respective condition (Wald test with Benjamini-Hochberg (BH) multiple test correction, padj <0.05, n = 3 donors per KO). The horizontal annotation bars on the top of the figure represent the stimulation condition (dark grey for resting and light for restimulated) and the vertical annotation bars represent the effect of the regulator in the IL-2Rα screens (colored boxes = statistically significant FDR < 0.05, navy = positive regulator of IL-2Rα and gold = negative regulator of IL-2Rα). Source Data

**Extended Data Fig. 6:**
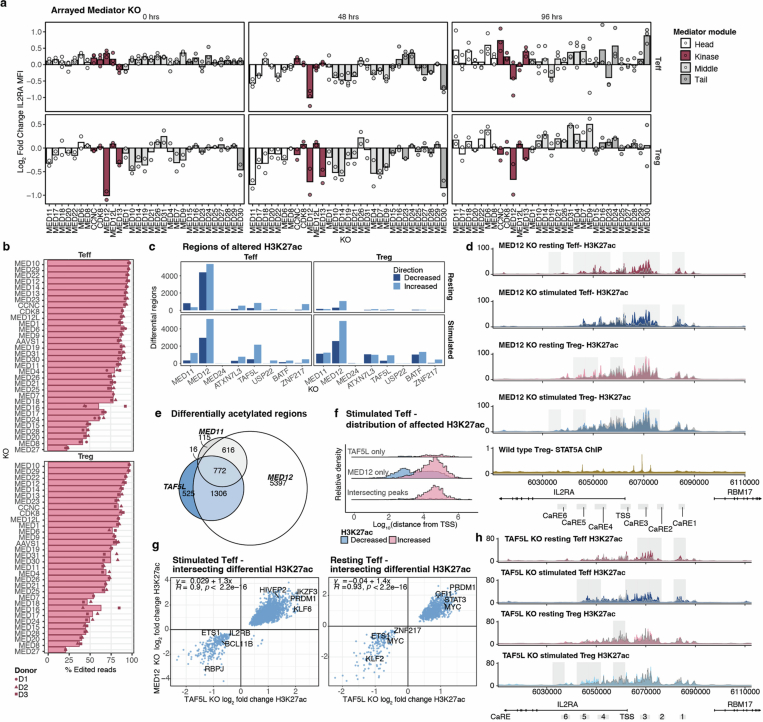
Mediator and SAGA complexes shape context-dependent regulation of IL-2Rα. **a**. Mediator complex subunit KO effects on expression of IL-2Rα as quantified by flow cytometry across contexts. Each color represents a Mediator complex structural module (n = 3 donors for Teffs and 2 donors for Tregs x 1 sgRNA per condition). **b**. Amplicon-seq genotyping of arrayed validation KOs to confirm editing. The mean percent modified (edited) reads as quantified by NGS is depicted for each of the targeted genes (Teff genotyping n = 3 donors, Tregs genotyping n = 2 donors). **c**. Select regulators of IL-2Rα affect H3K27ac distribution in CD4 + T cells. The number of differentially H3K27 acetylated regions in each KO cell population compared to control *AAVS1* KO cells is displayed on the y axis (Two-tailed Wald test with BH multiple test correction, padj <0.05, n = 2 donors per KO). The direction in which H3K27ac was altered is depicted as the color of the bar. **d**. Trackplot of the *IL2RA* locus depicting regions of differential acetylation between the MED12 KO (solid color) and *AAVS1* control KO (grey transparent) conditions from a representative donor. Light grey boxes distinguish regions of significantly differential acetylation between the MED12 KO and the *AAVS1* KO (Two-tailed Wald test with BH multiple test correction, padj <0.05, n = 2 donors per KO). *IL2RA* CaRE enhancer elements^6^ are annotated in grey boxes below gene tracks. STAT5A ChIP data sourced from public data (Methods). **e**. Venn diagram depicting differentially acetylated regions (relative to *AAVS1* KO control cells) for MED12, TAF5L, and MED11 KO samples. Differentially acetylated regions determined as described in c. **f**. Histogram depicting the distribution of differentially acetylated regions based on distance to the transcription start site of the nearest gene. The peak height is the proportional to the number of differentially acetylated regions across the samples. **g**. Correlation of TAF5L and MED12 differentially acetylated regions. Regions as described in c. depicted as the log_2_ fold change acetylation for the respective perturbations. **h**. Trackplot of H3K27ac as described in d for TAF5L KO instead of MED12 KO. Source Data

MED12 is part of the kinase domain of Mediator, which can function as an inhibitory component because its presence prevents binding of the complex to RNA polymerase II^27–29^. We perturbed one subunit from each functional Mediator module and performed bulk RNA-seq in resting and stimulated T_reg_ and T_eff_ cells. Much like the screen, MED12 and core Mediator knockouts often shared the same direction of effect, reflected by a positive correlation between MED12-regulated genes and those genes regulated by core subunits MED11, MED14 and MED31 (Fig. 4e and Extended Data Fig. 5a,b). We also compared the effects of different Mediator component knockouts on surface protein levels of IL-2Rα. Here we noted that MED12-knockout effects could be stronger and even partially discordant with other Mediator subunit knockouts, depending on the cell-type and stimulation context (Extended Data Fig. 6a,b). Collectively, these data reveal partially shared effects by MED12 and core Mediator, and depict MED12 as particularly important to promote expression of context-specific regulators of CD4^+^ T cell state.

We next assessed the effect of ablating key IL-2Rα regulators on the chromatin landscape at the *IL2RA* locus. H3K27ac is a mark of active enhancers and varies considerably between cell types and states^30^. We performed H3K27ac CUT&RUN following knockout of genes encoding select context-specific regulators: Mediator subunits (MED12, MED11 and MED24), SAGA subunits (TAF5L, ATXN7L3 and USP22), BATF and ZNF217. Perturbation of several regulators, especially MED12, resulted in significant changes in acetylation compared with *AAVS1*-knockout samples (Extended Data Fig. 6c). Downstream of the *IL2RA* transcription start site (TSS) showed significantly less levels of H3K27ac in MED12-knockout samples, specifically in T_reg_ cells. MED12-knockout T_eff_ cells had increased levels of acetylation in a region upstream of the TSS that is normally more acetylated in T_reg_ cells, which we previously characterized as a T_reg_-specific element called CaRE3 (ref. ^6^) (Extended Data Fig. 6d). T_reg_ chromatin immunoprecipitation followed by sequencing (ChIP–seq) data^31^ contained prominent STAT5A peaks within CaRE3, suggesting that increased STAT5 signalling could contribute to more T_reg_-like gene expression in MED12-knockout resting T_eff_ cells (Extended Data Fig. 6d). We observed that genome-wide regions of differential acetylation in the TAF5L knockout were highly correlated with MED12, including increased acetylation in T_eff_ cells at the *IL2RA* CaRE3 locus, suggesting a possible shared downstream regulator (Extended Data Fig. 6e–h). Collectively, these changes demonstrate a loss of context-specific chromatin features required for cell identity and state dynamics as the result of regulator perturbations.

## MED12 shapes chromatin at core genes

**Fig. 5:**
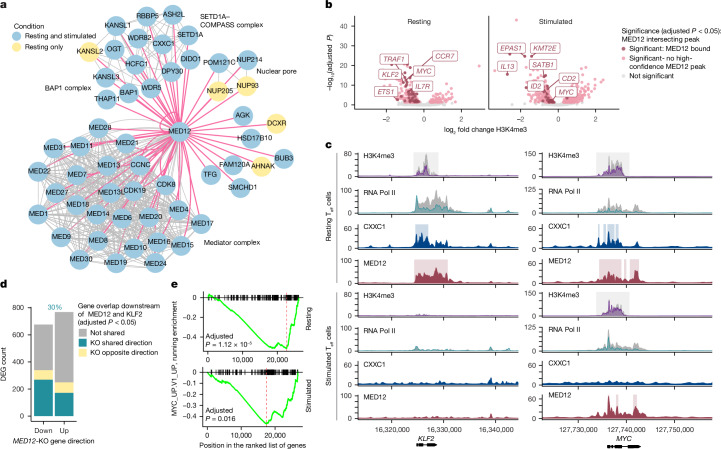
MED12 shapes chromatin landscapes to promote cell-type-specific and stimulation-specific regulation. **a**, Proteins enriched in CD4^+^ T_eff_ MED12 IP-MS with more than 100-fold enrichment relative to IgG control in one or more conditions (Bayesian FDR ≤ 0.05; *n* = 3 donors). The pink lines indicate enrichment in immunoprecipitation, and the grey lines are reported physical interactions in the STRING database. **b**, Gene loci with H3K4me3 altered by MED12-knockout relative to *AAVS1*-knockout control T_eff_ cells determined by CUT&RUN (*n* = 3 donors per condition). Significant regions intersecting MED12 high-confidence ChIP–seq peaks (Methods; *n* = 2 donors per condition) are coloured in red with select genes labelled. **c**, *KLF2* and *MYC* loci depicting differential H3K4me3 and RNA polymerase (Pol) II C-terminal domain (CTD) occupancy between the MED12-knockout (purple and turquoise) and *AAVS1*-knockout (grey) conditions from a representative donor. The light grey boxes indicate the region of differential H3K4me3 between the MED12 knockout and *AAVS1* knockout (adjusted *P* < 0.05; *n* = 3 donors). The coloured boxes indicate CXXC1 peaks and MED12 high-confidence peaks in *AAVS1*-knockout T_eff_ cells (*n* = 2 donors). **d**, Differentially expressed genes (DEGs) downstream of MED12 and KLF2 in resting CD4^+^ T_eff_ cells as described in Fig. 4c. KLF2-regulated genes are from Freimer et al.^19^ (adjusted *P*). **e**, Gene set enrichment analysis with Benjamini–Hochberg multiple test correction depicting significantly reduced enrichment of MYC signature genes (MYC_UP.V1_UP from msigdb) in MED12-knockout cells versus *AAVS1*-knockout control cells. Source Data

**Extended Data Fig. 7:**
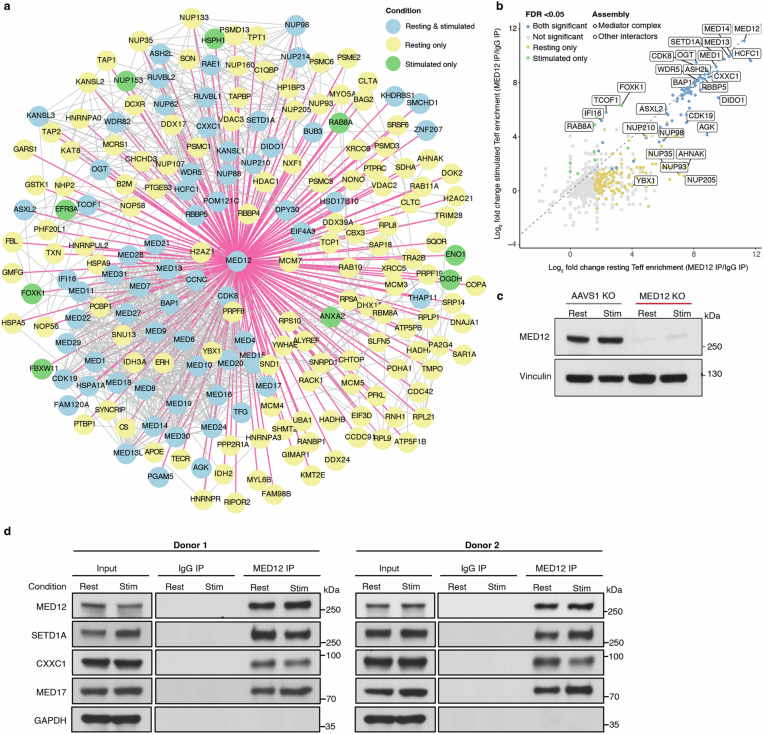
SETD1A/COMPASS complex members are enriched within an extensive MED12 protein interaction network within CD4+ Teffs. **a**. MED12 interaction partners in CD4+ Teffs. Proteins enriched in MED12 immunoprecipitation mass spectrometry (IP-MS) relative to IgG control (BFDR < = 0.05, n = 3 donors). Pink lines indicate enrichment in immunoprecipitation and grey lines are derived from reported physical interactions in STRING database^64^. **b**. Proteins from a. plotted by log_2_ fold change enrichment demonstrate high representation of SETD1A/COMPASS members. **c**. Western blot confirmation of MED12 protein loss with MED12 KO sgRNA s2770 targeting MED12 using antibody clone D9K5J used in IP experiments. Two donors were processed in one experiment. For gel source data, see Supplementary Fig. 1. **d**. MED12 IP validation of select interactors by western blot. Western blot of IgG and MED12 IP samples demonstrating interaction between MED12 and SETD1A, CXXC1, and MED17 in resting and stimulated Teffs in two human donors. For gel source data, see Supplementary Fig. 1.

To probe the mechanism of context-specific gene regulation by MED12 in human CD4^+^ T cells, we sought to define its interaction partners. MED12 lacks a DNA-binding domain and enzymatic function but possesses several intrinsically disordered regions ideal for protein–protein interactions. We performed endogenous immunoprecipitation mass spectrometry (IP-MS) of MED12 in resting and stimulated T_eff_ cells and identified 203 significant interaction partners across conditions (Bayesian FDR ≤ 0.05), including all members of the Mediator complex, except MED25, MED26 and MED12L (Extended Data Fig. 7a and Supplementary Table 4). The subset of proteins with over 100-fold enrichment in the MED12 pull down included numerous members of COMPASS, a histone-methylating complex (Fig. 5a and Extended Data Fig. 7b). Although COMPASS has several configurations, one particular assembly was represented including SETD1A, an H3K4me1–3 methyltransferase, and CXXC1, a DNA-binding protein. Western blotting further confirmed co-precipitation of SETD1A and CXXC1 with MED12 (Extended Data Fig. 7c,d).

**Extended Data Fig. 8:**
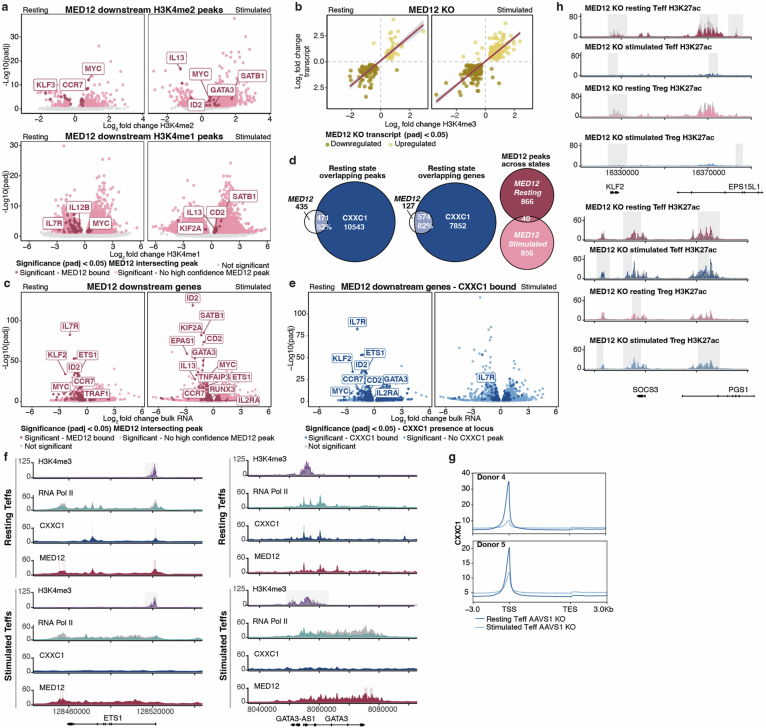
MED12 ablation disrupts chromatin at loci encoding regulators of rest and activation. **a**. Sites of H3K4me2/1 altered by MED12 KO relative to *AAVS1* KO Teffs (n = 3 donors per condition) determined by CUT&RUN. Significant regions intersecting high-confidence MED12 peaks, assessed by ChIP-seq (Methods, n = 2 donors per condition), are colored in red with select genes labeled. **b**. Correlation between genes with differential H3K4me3 and transcript expression comparing MED12 KO to *AAVS1* KO Teffs. **c**. Genes differentially expressed in MED12 KO relative to *AAVS1* KO (n = 3 donors per condition) colored as in a. **d**. Intersection of MED12 high-confidence peaks and CXXC1 peaks (left plot) and MED12 high-confidence bound genes and CXXC1 bound genes (middle plot) in resting Teffs. Intersection of MED12 high-confidence peaks between resting and stimulated states (right plot, n = 2 donors per condition). **e**. Genes differentially expressed in MED12 KO relative to AAVS1 KO Teffs. CXXC1 bound genes determined by ChIP-seq (Methods, n = 2 donors per condition) are colored in dark blue with select genes labeled. **f**. Trackplots of *ETS1* and *GATA3* loci depicting differential H3K4me3 and RNA Pol II CTD occupancy between the MED12 KO (purple and turquoise) and *AAVS1* KO control (grey) conditions from a representative donor. Light grey boxes define significantly differential H3K4me3 peaks comparing MED12 KO and *AAVS1* KO control (padj <0.05, n = 3 donors). Colored boxes indicate CXXC1 peaks and MED12 high-confidence peaks (padj <0.05, n = 2 donors, Methods). **g**. CXXC1 binding distribution at expressed genes in CD4+ Teffs determined via ChIP-seq. **h**. Trackplots of *KLF2* and *SOC3* loci depicting regions of differential H3K27ac between the MED12 KO (solid color tracks) and *AAVS1* control KO (grey transparent tracks) conditions from a representative donor. Light grey boxes distinguish regions of significantly differential acetylation between the MED12 KO and the *AAVS1* KO control (padj <0.05, n = 2 donors). Source Data

The interaction of MED12 with COMPASS led us to suspect that it may affect targeted H3K4 methylation. H3K4me1 and H3K4me2 are associated with poised and active enhancers, whereas H3K4me3 is concentrated at active TSSs and has been shown to directly promote transcription^32–34^. CUT&RUN demonstrated widespread changes to H3K4me1–3 distribution in MED12-knockout T_eff_ cells compared with control cells (Fig. 5b and Extended Data Fig. 8a). Changes in H3K4me3 were correlated strongly with altered gene expression in the MED12-knockout samples (Extended Data Fig. 8b). We defined genes bound by MED12 across states using ChIP–seq in *AAVS1*-knockout T_eff_ cells, using MED12-knockout samples to establish the background, resulting in high-confidence MED12 peaks (Methods). We intersected these peaks with regions of differential methylation or gene expression in the MED12-knockout samples and found that bound regions were predominantly associated with decreased levels of H3K4me3 and reduced expression (Fig. 5b and Extended Data Fig. 8c). A number of IL-2Rα regulators and T cell rest maintenance and activation-promoting factors were among these genes, including *KLF2*, *MYC* and *ETS1* at rest and *MYC* and *SATB1* after stimulation, suggesting that MED12 directly promotes their conditional expression. Again, using ChIP–seq, we found that CXXC1 was also present at many of these loci in resting cells, intersecting 52% of MED12-bound regions and 82% of MED12-bound genes (Fig. 5c and Extended Data Fig. 8d–f). CXXC1 peaks were less abundant in stimulated samples, suggesting that the protein may be displaced following activation, at which point MED12 localization also changes (Extended Data Fig. 8d–g). H3K27ac was also affected at several regulators, including the *KLF2* locus in resting T_eff_ and T_reg_ cells (Extended Data Fig. 8h). Comprehensively, these data suggest that COMPASS and MED12 colocalize at several key activation-state regulatory genes where MED12 functions as a positive regulator of gene expression.

**Extended Data Fig. 9:**
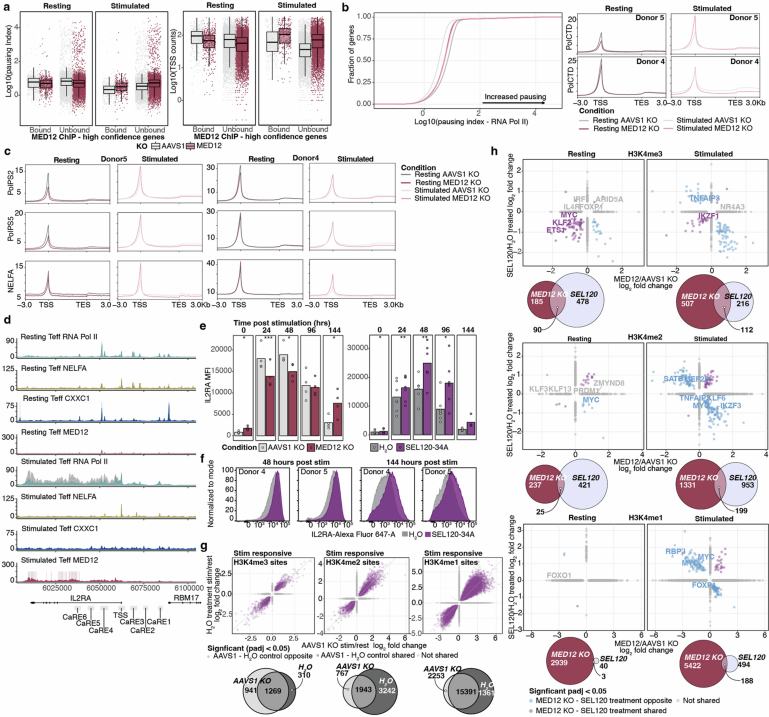
MED12 ablation results in widespread changes in polymerase pausing. **a**. Mean Pausing Index (left) and polymerase abundance at the TSS’s depicted for all genes expressed in Teffs (n = 2 donors per condition). Boxplots are separated by presence or absence of MED12 high-confidence peak(s) at the loci. Boxplot center line, median; box limits, upper and lower quartiles; whiskers, 1.5x interquartile range. **b**. Empirical cumulative distribution function plot displaying the mean pausing index for all genes expressed in Teffs (n = 2 donors per condition, left). RNA Pol CTD distribution across genes (right). **c**. Teff ChIP-seq binding distributions across genes expressed in Teffs (n = 2 donors per condition). **d**. Trackplot of the *IL2RA* locus depicting differences in occupancy of RNA Polymerase II CTD and NELFA in MED12 KO (turquoise and gold) and *AAVS1* control KO (grey tracks) conditions from a representative donor. Colored boxes indicate CXXC1 (blue) peaks and MED12 high-confidence peaks (red, Methods). **e**. IL-2Rα expression of CRISPR perturbed or kinase inhibitor (SEL120-34A) treated samples. Statistics performed using a paired two-tailed T test (MED12 KO: n = 4 donors, SEL120-34A: n = 7 donors all timepoints except T144 where n = 4 donors and T48 where n = 6 donors for SEL120-34A assay; MED12 KO assay: 0 hrs: p = 0.015, 24 hrs: p = 0.00013, 48 hrs: p = 0.025, 96 hrs: p = 0.75, 144 hrs: p = 0.02; SEL120-34A assay: 0 hrs: p = 0.045, 24 hrs: p = 0.002, 48 hrs: p = 0.0015, 96 hrs: p = 0.024, 144 hrs: p = 0.052). **f**. Representative flow plots from e. **g**. Stimulation responsive histone methylation shared between *AAVS1* KO control cells and vehicle-treated control cells. Top plot compares significantly differentially methylated sites in *AAVS1* KO resting vs stimulated Teffs (x axis) and vehicle (H_2_O)-treated resting vs stimulated Teffs (y axis). Euler plots below depict the overlap of all differentially methylated stimulation responsive sites displayed in the scatter plot (padj <0.05 significance threshold, n = 3 donors per condition). **h**. Top plot compares significantly differentially methylated sites in MED12 vs *AAVS1* KO Teffs (x axis) and SEL120-34A vs vehicle (H_2_O)-treated Teffs (y axis). Euler plots below depict the overlap of all differentially histone methylated sites displayed in scatter plot (padj <0.05, n = 3 donors per condition). Source Data

Loss of H3K4me3 has been associated with increased RNA polymerase II pausing, which results in an accumulation of polymerase at the TSS and coordinated loss in the gene body^33,34^. Using ChIP–seq in resting and stimulated T_eff_ cells, we found that pausing decreased globally following stimulation in *AAVS1*-knockout cells. However, MED12 ablation increased pausing in stimulated T_eff_ cells relative to *AAVS1*-knockout cells and decreased pausing in resting T_eff_ cells, resulting in diminished differences in pausing between states (Extended Data Fig. 9a,b). This effect is consistent with hyperactivation in rest and blunted stimulation response as we previously described. Accumulation of NELFA at the TSS of genes in stimulated MED12-knockout cells relative to *AAVS1*-knockout samples and decreased polymerase PS5 and PS2 in the gene body relative to the TSS provided additional evidence of increased pausing after activation, including at the *IL2RA* locus, which did not have significantly altered H3K4me3 (Extended Data Fig. 9c,d). In resting MED12-knockout cells, reduced polymerase at the TSS was a prominent global feature, suggesting inhibited recruitment (Extended Data Fig. 9a). Collectively, reduced pausing at rest and increased pausing after stimulation was a genome-wide trend for MED12-knockout cells.

Although global changes in polymerase activity reflect the altered activation state of the knockout cells, core regulators of rest and activation bound by MED12 exhibited more consistent changes in transcription. The TSS of the rest maintenance factor *KLF2* had significantly reduced H3K4me3 and increased pausing at rest in MED12-knockout cells, deviating from the overall trend (Fig. 5c). *MYC*, which participates in maintenance of both rest and promotion of the activation response, had reduced H3K4me3 in both states and exhibited reduced polymerase recruitment at rest and increased pausing following stimulation. The resulting strong downregulation of these genes following MED12 ablation (Fig. 4c) suggests a model in which MED12 drives expression of context-specific regulators. To assess the significance of these particular loci, we looked for evidence of their role in broader MED12 signalling. Using bulk RNA-seq data in resting CD4^+^ T_eff_ cells, we found that ablation of KLF2 alone was able to account for 30% of differentially expressed genes downstream of MED12 (Fig. 5d). In lieu of bulk RNA-seq for MYC-knockout T cells, we found that a strong negative enrichment of MYC overexpression induced genes in both resting and stimulated MED12-knockout samples (Fig. 5e). Of note, MYC has been characterized as a pause release factor and may contribute to global changes following activation^35^. Although we did not exhaust the important factors downstream of MED12 that probably contributed to its effects, we demonstrated that by promoting expression of several core regulatory factors, MED12 is able to establish central governance over broad regulatory networks.

We next asked whether the Mediator kinase CDK8 and the homologue CDK19 participate in MED12-driven regulation of activation. We used SEL120-34A, which is an inhibitor specific to CDK8 and CDK19. Treatment was sufficient to reproduce elevated levels of IL-2Rα in resting T_eff_ cells; however, when the cells were stimulated, IL-2Rα remained significantly higher than vehicle (H_2_O) control-treated cells, whereas MED12-knockout T_eff_ cells had lower IL-2Rα expression than *AAVS1*-knockout cells (Extended Data Fig. 9e,f). Next, we assessed changes in H3K4me1–3 following kinase inhibition. We first confirmed that the vehicle-treated control cells responded to stimulation similarly to *AAVS1*-knockout cells, finding high correlation between stimulation-responsive sites (Extended Data Fig. 9g). However, differentially methylated sites between SEL120-34A and vehicle-treated cells were poorly correlated with MED12-knockout effects, with the notable exception of shared reduced H3K4me3 at *KLF2*, *MYC* and *ETS1* in the resting condition (Extended Data Fig. 9h). Reduced expression of the gene encoding the resting-state maintenance factor KLF2 is consistent with elevated levels of IL-2Rα observed in the kinase inhibitor-treated cells. Collectively, these data suggest a complex role for the Mediator kinase, which contributes to the regulation of several rest maintenance factors, but does not explain the MED12-mediated regulation of stimulation responses.

## MED12 knockout limits activation-induced death

**Fig. 6:**
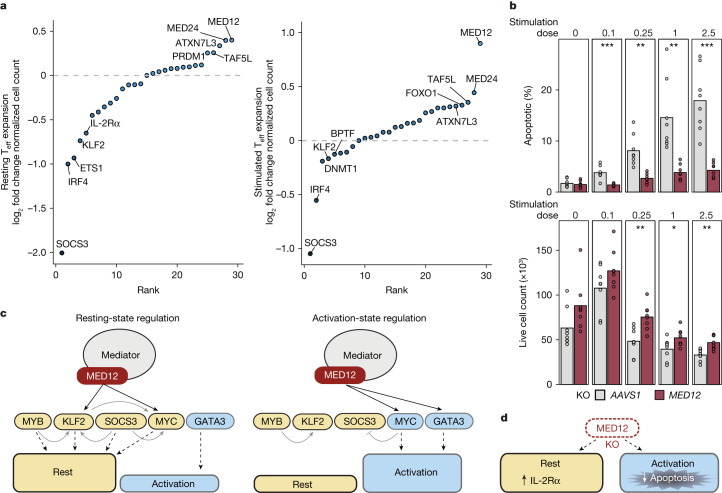
MED12 ablation limits activation-induced T cell apoptosis. **a**, Total cell abundance for each gene knockdown within the indicated Perturb-seq pool of single cells normalized using the sgRNA distribution in the plasmid library and represented as the log_2_ fold change compared with non-targeting cells (dashed line). **b**, Percentage of apoptotic cells and live T_eff_ cell count following various dosages of anti-CD3–CD28–CD2 stimulation reported relative to the manufacturer recommended dose. Two-tailed *t*-test comparing groups (*n* = 4 donors × 2 sgRNAs per target gene; for apoptosis, dose 0: *P* = 0.56, 0.1: ***P = 0.00087, 0.25: **P = 0.0011, 1: **P = 0.003 and 2.5: ***P = 0.00032; for live counts, dose 0: *P* = 0.68, 0.1: *P* = 0.14, 0.25: **P = 0.0026, 1: *P = 0.036 and 2.5: **P = 0.0017). **c**, Model of core regulatory networks controlling T cell rest and activation, both coordinated by MED12. The solid lines indicate regulatory effects on other factors, the dashed lines represent effects on overall states, and the solid black lines indicate potential direct regulation by MED12 as supported by ChIP–seq data. **d**, Phenotypic effects of MED12 ablation in CD4^+^ T_eff_ cells. Dashed lines represent the effects on overall states. Schematics in panels **c**,**d** were created using BioRender (https://biorender.com). Source Data

**Extended Data Fig. 10:**
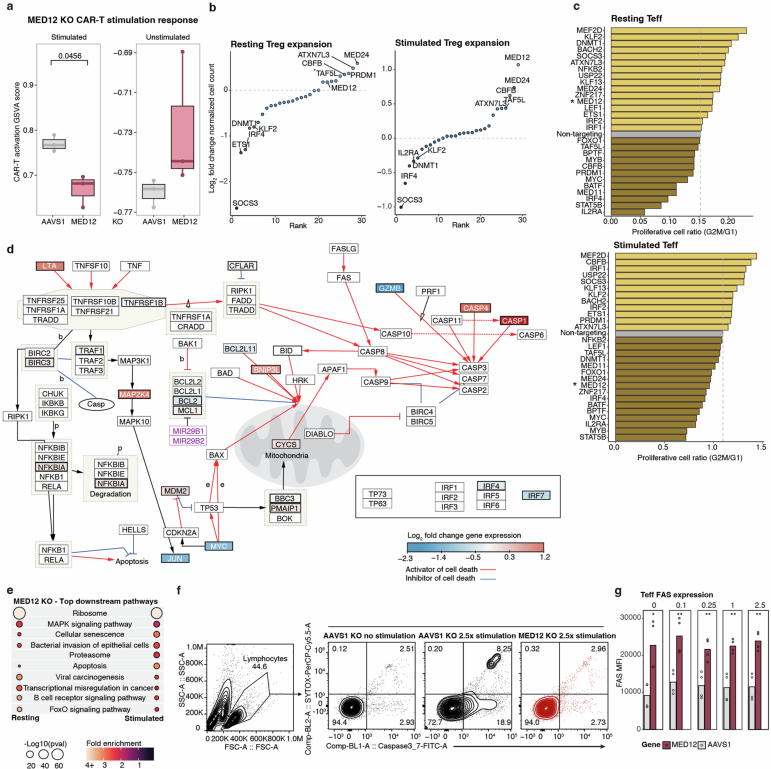
Activation-induced cell death pathways are dysregulated in MED12 KO T cells. **a**. MED12 KO CAR-T gene expression activation scores based on gene expression signature analysis of RNAseq data from Freitas et al. (GEO series GSE174279). The GSVA activation score for each group of samples is compared using a two-tailed T test and the Bonferroni adjusted p value displayed for significantly different groups. Boxplot center line, median; box limits, upper and lower quartiles; whiskers, 1.5x interquartile range (n = 3 donors per KO). **b**. Rank plot of cell abundance within the Perturb-seq single cell pools for each CRISPRi gene target for resting and stimulated Tregs. Cell abundance normalized by the sgRNA distribution in the plasmid library and represented as the log_2_ fold change compared to non-targeting control cells. The dashed line indicates the abundance of non-targeting control cells. **c**. Proliferative cell ratios assessed by gene signature of cycling cells within each CRISPRi regulator knockdown condition in the Perturb-seq pool. The estimated ratio of G2M/G1 cells within each condition is represented on the x axis. * Used to denote MED12. **d**. Differentially expressed genes (padj <0.05) from stimulated Teff MED12 KO vs *AAVS1* KO control cell bulk RNAseq data are illustrated on a schematic of the Apoptosis-Homo sapiens pathway from WikiPathways. **e**. Top pathways affected by MED12 KO in bulk RNAseq data. Apoptosis pathway genes were dysregulated by MED12 KO in stimulated Teffs. **f**. Flow cytometry gating strategy for activation-induced cell death (AICD) apoptosis assays. **g**. FAS cell surface expression in MED12 KO and *AAVS1* KO control cells (FAS MFI as quantified by flow cytometry is compared across perturbation conditions). Stars indicate significantly different MFI in MED12 KO cells compared to control *AAVS1* KO cells using a two-tailed T test (n = 2 donors x 2 sgRNAs per gene; Dose 0: p = 0.019, 0.1: p = 0.0071, 0.25: p = 0.0056, 1: p = 0.0047, 2.5: p = 0.0018). Source Data

Synthetic perturbation of key regulators is a promising strategy to improve adoptive T cell therapies. Recently, MED12 knockout was nominated by a genome-wide CRISPR screen in CAR-T cells to promote fitness. Ablation of MED12 resulted in improved CAR-T cells with sustained expansion and tumour control in preclinical models^36^. We speculated that an unreported but critical part of the therapeutic success of these experiments may be mediated by altered activation-state transitions, avoiding complete rest and a state of peak activation. Using bulk RNA-seq data from Freitas et al.^36^, we generated an activation score using genes upregulated in control CARs after stimulation. We then applied this score to the control and MED12-knockout CARs and found a significant decrease in activation for the stimulated MED12-knockout CARs compared with the controls (Extended Data Fig. 10a). Within our Perturb-seq pool, MED12-targeted cells experienced the largest increase in total T_eff_ cell counts, especially stimulated cells (Fig. 6a). Stimulated MED12-targeted T_reg_ cells also exhibited similar effects (Extended Data Fig. 10b). We asked why MED12-targeted cells with reduced activation capacity would be more abundant than non-targeting controls. On the basis of cell-state signatures in Perturb-seq, MED12-targeted cells showed a slight increase in the proportion of proliferative cells in the resting condition but a substantial decrease in the proportion of proliferative cells in the stimulated condition compared with non-targeting cells (Extended Data Fig. 10c). Across the perturbed T cell pool, the percentage of proliferative cells and total cell abundance were not well correlated, possibly due to decreased viability.

We reasoned that the reduced stimulation responses in MED12-targeted cells may instead improve cell durability by limiting activation-induced cell death. Consistent with this hypothesis, genes associated with ‘apoptosis’ were enriched among genes differentially expressed between MED12-knockout and *AAVS1*-knockout cells, driven by a mix of both upregulated and downregulated genes (Extended Data Fig. 10d,e and Supplementary Table 5). To determine whether MED12 knockout altered apoptosis in response to stimulation (activation-induced cell death), we performed a dose–response of stimulation strength using anti-CD3–CD28–CD2 soluble tetramers and quantified apoptosis via caspase-3/7 activation. As expected, apoptosis increased with stimulation dose in *AAVS1*-knockout control T_eff_ cells (Fig. 6b and Extended Data Fig. 10f). By marked contrast, MED12-knockout cells underwent minimal apoptosis in response to even strong stimulation. The apoptosis-initiating receptor FAS was elevated on MED12-knockout cells throughout the assay, which was possibly the result of an affected feedback loop (Extended Data Fig. 10g). The MED12-knockout-associated reductions in apoptosis translated to improved live cell abundance, providing an explanation for improved cell durability following MED12 ablation in the stimulated condition (Fig. 6c,d).

## Discussion

CRISPR screens in multiple primary cell conditions collectively defined a dynamic network of *trans*-regulators that enable cell-type-specific and state-specific expression of IL-2Rα. We uncovered marked differences in regulation between T_reg_ cells with constitutively high levels of IL-2Rα and stimulated T_eff_ cells with transiently high levels. T_eff_ cells utilize waves of regulators to maintain rest (KLF2 and MED12), achieve peak expression (GATA3 and MED12) and return to a resting state (TAF5L and SOCS3), whereas T_reg_ cells appear to utilize a more static but expansive network of regulators to maintain IL-2Rα levels. One key insight that emerged is that the resting state depends on multiple rest maintenance factors that form an overwhelmingly positive-regulatory network, collectively promoting expression of a core rest factor KLF2. Activation-promoting factors repress this rest maintenance network following stimulation to achieve a maximal activated cell state. MED12 serves as a dynamic regulator of state-specific gene expression via orchestration of downstream factors across these networks. Mediator accumulation is a feature of super-enhancers and has been associated with their activity in diverse cell types, suggesting that MED12 is recruited to the locus of key regulatory genes that are under precise enhancer regulation^37,38^.

Multiple studies have suggested that cycles between T cell rest and activation can influence the durability of adoptive T cell therapies^39,40^. Our findings suggest that loss of MED12 tunes T cell activation responses and limits activation-induced apoptosis to improve durability. This resistance to apoptosis appears to function independent of FAS (which was upregulated following MED12 ablation), another pathway that has been targeted to enhance cell therapy persistence^41^. Targeting MED12 and other context-dependent regulators may offer additional advantages by enhancing the fitness of T_eff_ cells, but reducing IL-2Rα expression and the suppressive capacity of T_reg_ cells (cells that could limit the efficacy of bulk CAR-T products^36^). Increased expression of IL-2Rα by resting T_eff_ cells and decreased expression by T_reg_ cells may help T_eff_ cells to compete more effectively for IL-2.

Here we limited our study to *trans*-regulators within the CD4^+^ T cell compartment to comprehensively profile T_eff_ cells and T_reg_ cells across stimulation conditions, but additional screening conditions, perturbations and phenotypic readouts would provide further insight into context-specific gene regulatory networks. The current work revealed the architecture of the gene regulatory networks controlling dynamic expression of IL-2Rα across cell types and activation states. More broadly, it provides fundamental insights into the regulation of T cell identity and mechanisms governing transitions between rest and activation.

## Methods

### Primary human T cell isolation and expansion

CD4^+^ regulatory and effector T cells were isolated from fresh peripheral blood Leukopaks (70500, STEMCELL Technologies) from healthy human donors with institutional review board-approved informed written consent (STEMCELL Technologies). The contents of the Leukopaks were washed twice with a 1X volume of EasySep buffer (DPBS, 2% FBS and 1 mM EDTA (pH 8.0)) using centrifugation. The washed cells were resuspended at 200 × 10^6^ cells per millilitre in EasySep buffer and isolated with the EasySep Human CD4^+^CD127^low^CD25^+^ Regulatory T Cell Isolation Kit (18063, STEMCELL Technologies), according to the manufacturer’s protocol. Following isolation with the kit, T_reg_ cells were stained Alexa Fluor 647 anti-human IL-2Rα antibody (302618, BioLegend; diluted 1:25), phycoerythrin anti-human CD127 (557938, Beckon Dickinson; diluted 1:50) and Pacific Blue anti-human CD4 antibody (344620, BioLegend; diluted 1:50) and isolated with FACS performed on a BD FACS ARIA Fusion 1 (656700) to ensure a pure population without contaminating effector cells. After sorting pure CD4^+^CD127^low^CD25^+^ T_reg_ cells, the cells were seeded at 1 × 10^6^ cells per millilitre in XVIVO-15 (02-053Q, Lonza) supplemented with 5% FCS, 55 µM 2-mercaptoethanol, 4 mM *N*-acetyl l-cysteine and 200 U ml^−1^ IL-2 (10101641, Amerisource Bergen). T_eff_ cells were seeded at 1 × 10^6^ cells per millilitre in RPMI-1640 supplemented with 10% FCS, 2 mM l-glutamine (25030081, Fisher Scientific), 10 mM HEPES (H0887-100ML, Sigma), 1X MEM non-essential amino acids (11140050, Fisher), 1 mM sodium pyruvate (11360070, Fisher Scientific), 100 U ml^−1^ penicillin–streptomycin (P4333-100ML, Sigma) and 50 U ml^−1^ IL-2 (10101641, Amerisource Bergen). Both cell subsets were then stimulated with ImmunoCult human CD3/CD28/CD2 T cell activator (10990, STEMCELL Technologies) at 25 µl ml^−1^ for T_reg_ cells and 6.25 µl ml^−1^ for T_eff_ cells. Cells were cultured at 37 °C with 5% CO_2_. Following activation and electroporation, cells were split 1:2 every 48 h to maintain an approximate density of 1 × 10^6^ cells per millilitre and supplemented with respective doses of IL-2.

### Pooled CRISPR knockout screen *trans*-regulator editing

Pooled screens were performed following the protocol described previously^19^. In brief, 24 h after stimulating and plating the T cells, the *trans*-regulator lentiviral library^19^ was added to each culture (Supplementary Table 6). The cells were counted before transduction, and virus was added at a multiplicity of infection of 0.8, using gentle mixing to disperse the viral media without disrupting cell bundling. The cells were then incubated at 37 °C for an additional 24 h, pelleted by centrifugation, and viral media were replaced with fresh media supplemented with IL-2.

Twenty-four hours after washing, the cells were pelleted by centrifugation at 150*g* for 10 min, resuspended at 1.5 × 10^6^ cells per 17.8 µl supplemented with P3 Primary Cell Nucleofector Buffer (component of V4SP-3960, Lonza) and combined with 7.2 µl ribonucleoprotein particle (RNP)/1.5 × 10^6^ cells in a sterile 10-ml reservoir. After mixing the cells and RNPs, 25 µl of the mixture was distributed to the wells of a 96-well Nucleocuvette Plate (component of V4SP-3960, Lonza). Cells were nucleofected using code EO-115 for T_reg_ cells and EH-115 for T_eff_ cells on the Lonza 4D-Nucleofector System with the 96-well Shuttle. Immediately after nucleofection, 90 µl pre-warmed cell-appropriate medium was added to each well, and the cells were incubated at 37 °C for 15 min. Following incubation, cells were seeded at 1 × 10^6^ cells per millilitre in media supplemented with IL-2.

### IL-2Rα screen sorting and library preparation

Transduced and electroporated cells were expanded for a minimum of 6 days following editing before sorting. Cell sorting was performed 10 days following isolation for the resting screens. For the stimulated T_eff_ screen, cells were restimulated with ImmunoCult human CD3/CD28/CD2 T cell activator (10990, STEMCELL Technologies) 9 days following initial isolation, and sorting was performed 72 h after restimulation, at the time of peak IL-2Rα expression. Before sorting, cells were counted, washed once with EasySep buffer and stained with Alexa Fluor 647 anti-human IL-2Rα antibody (302618, BioLegend; diluted 1:25). Cells were then washed and resuspended in EasySep buffer. During sorting, cells were gated on the GFP^+^ population (lentiviral sgRNA library marker) and the top and bottom 20% of IL-2Rα-expressing cells were sorted into 15-ml conical tubes coated with FCS. Isolated cells were pelleted, counted and lysed. Genomic DNA extraction was performed using phenol-chloroform extractions, and sgRNA libraries were amplified and prepared for sequencing using custom primers. Libraries were sequenced on an Illumina HiSeq 4000 at the UCSF CAT.

### Screen analysis

All pooled screens were analysed with MAGeCK^42^ (v0.5.9.5). MAGeCK count was performed on all donors using --norm-method none followed by MAGeCK test --sort-criteria pos to identify genes that resulted in a statistically significant change in IL-2Rα expression. Results are calculated as the IL-2Rα^low^ bin/IL-2Rα^high^ bin. Screen visualization is represented as the IL-2Rα^high^ bin/IL-2Rα^low^ bin by flipping the sign for the fold change. All genes with an FDR-adjusted *P* < 0.05 were considered significant.

### Arrayed CRISPR knockout of select regulators

Guide-loaded Cas9 RNPs were assembled with custom CRISPR RNAs (crRNAs) (Dharmacon), which were resuspended in IDT duplex buffer (11-01-03-01, IDT) at 160 µM. Sequences are provided in Supplementary Table 6. Dharmacon Edit-R CRISPR–Cas9 synthetic tracrRNA (U-002005-20, Dharmacon) also resuspended in nuclease-free duplex buffer at 160 µM was combined at a 1:1 molar ratio in a 96-well plate and incubated at 37 °C for 30 min. Single-stranded donor oligonucleotides (sequence: TTAGCTCTGTTTACGTCCCAGCGGGCATGAGAGTAACAAGAGGGTGTGGTAATATTACGGTACCGAGCACTATCGATACAATATGTGTCATACGGACACG; 100 µM stock) was added to the complex at a 1:1 molar ratio and incubated at 37 °C for 5 min. Finally, Cas9 protein (MacroLab; 40 µM stock) was added at a 1:2 molar ratio and incubated at 37 °C for 15 min. The resulting RNPs were frozen at −80 °C until the day of electroporation and were thawed to room temperature before use. Forty-eight hours following T cell activation, the cells were pelleted at 100*g* for 10 min and resuspended in room temperature P3 Primary Cell Nucleofector Buffer (V4XP-3032, Lonza) at 1.5 × 10^6^ cells per 17.8 µl. Cells (1.5 × 10^6^) were transferred to each RNP-containing well and mixed gently. Of the combined RNP cell solution, 25 µl was transferred to a 96-well electroporation cuvette plate (VVPA-1002, Lonza) and nucleofected with pulse code DS-137. Immediately following electroporation, the cells were gently resuspended in 90 µl warmed media and incubated at 37 °C for 15 min. After recovery, the cells were cultured in 96-well round-bottom plates at 1 × 10^6^ cells per millilitre for the duration of the experiment. To prevent edge effects, the sgRNAs were randomly distributed across each plate, and the first and last columns and rows of each plate were filled with PBS to prevent evaporation. Unless otherwise specified, CRISPR–Cas9-edited cells were restimulated on day 8 following isolation for stimulation response arrayed assays with ImmunoCult human CD3/CD28/CD2 T cell activator (10990, STEMCELL Technologies).

### Genotyping of arrayed knockouts

On the final day of the respective assay, genomic DNA was isolated using DNA QuickExtract (QE09050, Lucigen) according to the manufacturer’s protocol. Primers were designed to flank each sgRNA target site. Amplicons of the region were generated by adding 1.25 µl each of forwards and reverse primer at 10 µM to 5 µl of sample in QuickExtract, 12.5 µl of NEBNext Ultra II Q5 master mix (M0544L, NEB) and H_2_O to a total 25 µl reaction volume. Touchdown PCR was used with the following cycling conditions: 98 °C for 3 min, 15 cycles of 94 °C for 20 s followed by 65 °C to 57.5 °C for 20 s (0.5 °C incremental decreases per cycle) and 72 °C for 1 min, and a subsequent 20 cycles at 94 °C for 20 s, 58 °C for 20 s and 72 °C for 1 min, and a final 10-min extension at 72 °C. Amplicons were diluted 1:200 and Illumina sequencing adapters were then added in a second PCR. Indexing reactions included 1 µl of the diluted PCR1 sample, 2.5 µl of each the forwards and the reverse Illumina TruSeq indexing primers at 10 µM each, 12.5 µl of NEB Q5 master mix and H_2_O to a total 25 µl reaction volume. The following PCR cycling conditions were used: 98 °C for 30 s, followed by 98 °C for 10 s, 60 °C for 30 s and 72 °C for 30 s for 12 cycles, and a final extension period at 72 °C for 2 min. Samples were pooled at an equivolume ratio and SPRI purified before sequencing on an Illumina MiSeq with PE 150 reads. Analysis was performed with CRISPResso2 (v2.2.7)^43^ CRISPRessoBatch --skip_failed --n_processes 4 --exclude_bp_from_left 5 --exclude_bp_from_right 5 --plot_window_size 10.

### Flow cytometry analysis of arrayed knockouts

The BioLegend FoxP3 Fix/Perm kit (421403, BioLegend) was used for staining according to the manufacturer’s protocol. Cells were washed in EasySep buffer before extracellular staining. Cells were stained with Alexa Fluor 647 anti-human IL-2Rα (CD25) antibody diluted 1:25 (302618, BioLegend), Ghost Dye Red 780 diluted 1:1,000 (13-0865-T500, Tonbo) and BV711 anti-human CD4 diluted 1:50 (344648, BioLegend) for 20 min at 4 °C and then washed once with EasySep buffer. After fixing and permeabilizing according to the kit, intracellular staining was performed with phycoerythrin anti-mouse/human Helios antibody (137216, BioLegend), KIRAVIA Blue 520 anti-human CD152 (also known as CTLA-4) antibody (349938, BioLegend) and Pacific Blue anti-human FOXP3 antibody (320116, BioLegend) each diluted 1:50 in permeabilization buffer for 30 min at room temperature. Cells were subsequently washed in permeabilization buffer and resuspended in EasySep buffer before running on the Thermo Fisher Attune NxT flow cytometer (A29004). Analysis of flow data was performed in FlowJo (v10.8.1). Gating was performed to select for lymphocytes, singlets, live cells (Ghost Dye negative) and CD4^+^ cells in the specified order. This population was then used to calculate the median fluorescence intensity for IL-2Rα or CTLA4. Visualization was performed in R using ggplot2 (v3.4.1).

### Cloning and lentivirus preparation

CRISPRi sgRNAs for Perturb-seq were selected from the Dolcetto library^44^ and cloned into the LGR2.1 plasmid backbone (Addgene #108098). A lenti EF1a-Zim-3-dCas9-P2A-BSD was generated using Gibson assembly as previously described^45^. Lentivirus was prepared according to the a previous protocol^25^.

### Perturb-seq

Twenty-four hours after stimulation of isolated human T_reg_ cells and T_eff_ cells from two donors, the cells were transduced with Zim3–dCas9 lentivirus at 3% v/v. The following day, Perturb-seq sgRNA library lentivirus was added at 0.75% v/v (multiplicity of infection of 0.3). Forty-eight hours after transduction with Zim3–dCas9, 10 mg ml^−1^ blasticidin (A1113903, Gibco) was added to each sample to select for dCas9^+^ cells. Blasticidin was replenished every 48 h until the cells were processed for sequencing. Eight days after initial isolation and stimulation of cells, half of the T_reg_ and T_eff_ cell culture was restimulated with ImmunoCult human CD3/CD28/CD2 T cell activator (10990, STEMCELL Technologies). On the tenth day after initial isolation, the resting and 48-h restimulated samples were collected for 10X single-cell sequencing. First, cells from each donor within the same stimulation and cell-type condition were pooled at equal concentrations. Sorting was performed to isolate live GFP^+^ cells from each condition. Sorted cells were processed according to the Chromium Next GEM Single Cell 5′ HT Reagent Kits v2 (Dual Index) with Feature Barcode technology for CRISPR Screening and Cell Surface Protein guide User Guide, CG000513. In brief, sorted cells were pelleted and washed once with cell staining buffer (420201, BioLegend). Next, the samples were blocked with Human TruStain FcX Fc Blocking reagent (422302, BioLegend). Meanwhile, TotalSeq-C Human Universal Cocktail V1.0 (399905, BioLegend) was prepared using cell staining buffer (420201, BioLegend), and TotalSeq-C0251 anti-human hashtag antibodies 1–4 (394661, BioLegend) were added to aliquots of the cocktail. After blocking, cells were stained with TotalSeq-C cocktail including one hashtag per cell and stimulation condition. After staining, the cells were washed three times in cell staining buffer. The samples were then resuspended in PBS with 1% BSA (Gibco) for final counting. The resulting samples were pooled across conditions and approximately 65,000 cells per well were loaded into eight wells of a Chromium Next GEM Chip N Single Cell Kit (1000375, 10X Genomics) for GEM generation. The samples were prepared for sequencing using the Chromium Next GEM (Gel Bead-in-emulsion) Single Cell 5′ HT Kit v2 (1000374), 5′ Feature Barcode Kit (1000256) and 5′ CRISPR Kit (1000451) according to the manufacturer’s protocol. GEM generation and library preparation were performed by the Gladstone Genomics Core. The resulting libraries were sequenced using a NovaSeqX Series 10B flowcell (20085595, Illumina) at the UCSF CAT.

### Perturb-seq analysis

Fastqs for each 10X well were concatenated across lanes and flow cells. Alignment of Perturb-seq data and count aggregation for the gene expression, CRISPR sgRNA and antibody-derived tag (ADT) libraries was performed with cellranger^46^ count (v7.1.0) using the default settings and –expect-cells=45000 –chemistry=SC5P-R2. Gene expression fastqs were aligned to ‘refdata-gex-GRCh38- 2020-A’ human transcriptome reference acquired from 10X Genomics. SgRNA sequences were aligned to a custom reference file using the pattern TAGCTCTTAAAC(BC), whereas ADTs were aligned to the TotalSeq-C-Human-Universal-Cocktail-399905-Antibody-reference-UMI-counting.csv provided by BioLegend, also including the hashtag oligo (HTO) sequences, which were used to distinguish each cell-type and stimulation condition. Counts for each respective library were aggregated across wells with cellranger aggr using the default settings. Cells were assigned to a donor using genetic demultiplexing with Souporcell^47^ (https://github.com/wheaton5/souporcell). For each well, souporcell_pipeline.py was run using the bam file and cellranger count output barcodes.tsv as input in addition to the reference fasta. Donor calls shared across wells were identified using shared_samples.py using the vcf file outputs from Souporcell.

Perturb-seq analysis was performed in R (v4.3.1) using Seurat^48^ (v4.3.0.1) based on code previously published^49^. Count matrices were imported into R using the Seurat Read10X function. After creating a Seurat object with CreateSeuratObject, quality filtering was performed to retain cells with more than 1,000 RNA features identified and less than 7.5% mitochondrial RNA. Cells without a singular donor assignment were also excluded from the object as well as cells with more than one HTO assignment as determined after running HTODemux. Low abundance transcripts were filtered using the threshold of ten cells per feature and TCR genes were removed from the primary RNA assay as they were found to be a major source of variance in the dataset. No sgRNA targets were removed as the number of cells in each condition exceeded the threshold set of 150 cells. After filtering, gene expression counts were normalized and transformed using the Seurat SCTransform function with regression of both S phase score and G2/M phase score, as described on Satija (https://satijalab.org/seurat/articles/cell_cycle_vignette.html). ADT counts were normalized using the centred log-ratio (CLR) normalization method of NormalizeData. After generating principal component analysis of both normalized and transformed RNA and ADT data, Harmony^50^ (v0.1.1) was used to correct for donor-associated variability in the dataset. The resulting normalized and transformed counts were used for downstream analysis unless otherwise specified. Uniform manifold approximation and projections (UMAPs) were generated using the transformed and corrected RNA and ADT counts with Seurat function FindMultiModalNeighbors followed by RunUMAP using weighted.nn. Before cell-type-specific analysis, T_reg_ cells were manually filtered to include only cells belonging to clusters with FOXP3 and IKZF2 expression to maximize cell purity (clusters 1, 7, 8, 15, 6, 4, 19, 20, 17 and 23).

Activation scoring was performed according to Schmidt et al.^25,49^. In brief, Seurat FindMarkers was used to identify differentially expressed genes between stimulated and resting non-targeting control cells within the T_eff_ cells and T_reg_ cells individually. Genes that had a log_2_-transformed fold change of more than 0.25 and were detected in 10% of restimulated or resting cells were used to generate gene weights for the score calculated as sum(GE × GW/GM), where GE is the normalized/transformed expression count of a gene, GW is the weight of the gene, and GM is the mean expression of the gene in non-target control cells of the respective cell type. Wilcoxon tests were performed to determine significance compared with non-targeting control cells with Bonferroni correction for multiple hypothesis testing (Supplementary Table 7). To observe the effect of each sgRNA within independent cell and stimulation conditions, the cells were subset by HTO. RNA and ADT normalization, transformation and donor variability correction were repeated for each subset as described above for the combined dataset. UMAPs were generated using the transformed and corrected RNA and ADT counts with Seurat function FindMultiModalNeighbors followed by RunUMAP using weighted.nn. Cell cycle quantification for each subset was performed using cycle assignments generated using the Satija cell cycle vignette referenced above.

Pseudobulking of resting and stimulated T_reg_ and T_eff_ cell samples was performed using Seurat AggregateExpression grouped by HTO, target gene and donor pulling from the counts slot (sgRNAs targeting the same gene were collapsed within the same donor). Differential expression analysis was performed with the resulting pseudobulked raw counts for both RNA and ADTs. DESeq2 (v1.32.0)^51^ was used to identify differentially expressed genes and proteins between each sgRNA and non-targeting control sample within each cell-type and stimulation condition, using donor information as a covariate. Network plots of differentially expressed gene connections were visualized in R using influential^52^ (v2.2.7) and ggraph^53^ (v2.1.0), including only genes with an adjusted *P* < 0.05. Other visualization of differentially expressed genes and surface proteins was performed using ggplot2 (v3.4.1).

### Bulk RNA-seq

At their respective timepoints, resting and 48-h restimulated cells were pelleted and resuspended at 1 × 10^6^ cells per 300 µl of RNA lysis buffer (R1060-1-100, Zymo). Cells were pipette mixed and vortexed to lyse and frozen at −80 °C until RNA isolation was performed. RNA was isolated using the Zymo-Quick RNA micro prep kit (R1051) according to the manufacturer’s protocol with the following modifications: after thawing the samples, each sample was vortexed vigorously to ensure total lysis before loading into the extraction columns. The optional kit provided DNAse step was skipped, and instead RNA was eluted from the isolation column after the recommended washes and digested with Turbo-DNAse (AM2238, Fisher Scientific) at 37 °C for 20 min. Following digestion, RNA was purified using the RNA Clean & Concentrator-5 kit (R1016, Zymo) according to the manufacturer’s protocol. The purified RNA was submitted to the UC Davis DNA Technologies and Expression Analysis Core to generate 3′ Tag-seq libraries with unique molecular indices (UMIs). Barcoded sequencing libraries were prepared using the QuantSeq FWD kit (Lexogen) for multiplexed sequencing on a NextSeq 500 (Illumina).

### Bulk RNA-seq analysis

RNA-seq data were processed using the pipeline previously described^19^. In brief, fastq adapter trimming was performed with cutadapt (v2.10). Low-quality bases were trimmed with seqtk (v0.5.0). Reads were then aligned with STAR^54^ (v2.7.10a) and mapped to GRCh38. UMI counting and deduplication was performed with umi_tools^55^ (v1.0.1) and gene counts were generated from the deduplicated reads using featureCounts (subread v2.0.1) using Gencode v41 basic transcriptome annotation. Quality control metrics were generated for each sample with Fastqc^56^ (v0.11.9), rseqc^57^ (v3.0.1) and Multiqc^58^ (v1.9). Differentially expressed genes between Mediator knockouts and *AAVS1*-knockout samples as well as stimulated and resting *AAVS1*-knockout samples (Supplementary Table 8) were identified from the deduplicated count matrix using DESeq2 (v1.32.0)^51^ in R (v4.1.0). Comparisons were made within each cell-type and stimulation condition across three donors, using donor ID as a covariate in the model. Normalized counts were generated using a DESeqDataSet containing all samples, followed by estimateSizeFactors and counts(normalized=TRUE). *AAVS1*-knockout normalized sample counts were then subset and averaged across donors for visualization.

Differentially expressed genes for MED12-knockout versus *AAVS1*-knockout samples were defined by a cut-off of adjusted *P* < 0.05 (Supplementary Table 3). Comparison of the effects of MED12-knockout differentially expressed genes across stimulation-responsive categories was performed by grouping MED12-knockout versus *AAVS1*-knockout differentially expressed genes according to their stimulation-responsive behaviour in control cells (stimulation response = adjusted *P* < 0.05 and abs(log_2_ fold change) > 1). The Bonferroni-adjusted *P* value resulting from a two-tailed *t*-test is displayed (Fig. 4a), comparing each stimulation-responsive group to the non-stimulation-responsive group. The boxplot centre line denotes the median; the box limits indicate the upper and lower quartiles; the whiskers denote the 1.5-times interquartile range (genes per group (downregulated, not stimulation responsive and upregulated) = resting T_eff_ cells: 272, 954 and 218; stimulated T_eff_ cells: 242, 1,432 and 467; resting T_reg_ cells: 269, 1,491 and 241; and stimulated T_reg_ cells: 245, 1,945 and 426).

A one-sided Fisher’s exact test for regulators of IL-2Rα within the differentially expressed genes downstream of MED12 was determined using screen results from the matched cell-type and stimulation conditions (Fig. 4b). Genes were subset to those targeted in the screen library and detected in CD4^+^ T cell bulk RNA-seq (genes per group: regulators, non-regulators = resting T_eff_ cells: 62 and 807; stimulated T_eff_ cells: 41 and 824; and resting T_reg_ cells: 82 and 787). Pathway analysis was performed using PathfindR^59^ (v1.6.4) including KEGG, Reactome and GO-BP gene sets and the lowest *P* value is displayed. Visualization was performed after removing KEGG disease pathways. Apoptosis pathway visualization was performed using Cytoscape^60^ (v3.8.2). Gene set enrichment analysis was performed with clusterProfiler^61^ (v4.10.1) using msigdbr (v7.5.1) on all human gene sets.

### SEL120-34A treatment

SEL120-34A (S8840, Selleckchem) was reconstituted in ultrapure H_2_O according to the manufacturer’s recommendations. Cells were treated every 48 h with a 1 µM dose, and treatment was started 48 h following cell isolation to align with the time at which cells are edited in CRISPR-based experiments. Restimulation of cells for flow cytometry and CUT&RUN was performed 10 days after initial isolation.

### Endogenous immunoprecipitation of MED12

Immunoprecipitation base buffer (0.05 M Tris-HCl pH 7.5, 0.15 M NaCl, 0.001 M EDTA and AP MS water) was prepared the day of the experiment. Of resting and 48-hour restimulated cells, 20 × 10^6^ cells per sample and immunoprecipitation were washed twice with PBS. Samples were then lysed in 500 µl lysis buffer per 10 × 10^6^ cells (Base buffer, 1X PhosphoStop (04906837001, Roche), 1X Complete mini-EDTA protease inhibitor cocktail tablets (11836170001, Sigma-Aldrich), 0.50% NP-40 Surfact-Amps Detergent Solution (85124, Thermo Scientific) and incubated on nutator for 30 min at 4 °C. To digest chromatin, tip sonication was performed in round with incubation on ice between each step: 7 s 12%, 7 s 12%, 7 s 12% and 7 s 15% with four rounds of sonication total. Cell lysate was clarified by centrifugation at 3,500*g* for 10 min at 4 °C. A bicinchoninic acid (BCA) assay was performed for each sample, and protein concentrations were normalized across conditions. Of whole-cell lysate, 10% was reserved for input, and samples were split into MED12 (14360, Cell Signaling Technologies) immunoprecipitation and rabbit IgG isotype control (3900, Cell Signaling Technologies) immunoprecipitation conditions. In each case, 10 µg antibody was added to a 1.5 ml protein lo bind tube containing clarified protein and samples were incubated overnight at 4 °C, with rotation on a nutator. In the morning, Pierce protein A + G magnetic beads (88802, Thermo Fisher) were washed four times using 1 ml of lysis buffer per 1 ml of bead slurry, allowing the beads to bind to a magnet between each wash before removing the buffer. After the final wash, beads were resuspended in lysis buffer at the original bead slurry volume, and 50 µl was added to each sample. The lysate–antibody–bead mixture was then incubated at 4 °C for 2 h with rotation on a nutator. After incubation, beads were bound to a magnetic tube rack and washed one time with immunoprecipitation buffer + NP-40 (immunoprecipitation buffer + 0.05% NP-40) followed by three washes with a 900 µl immunoprecipitation buffer. The resulting purified proteins were processed for mass spectrometry or western blot.

### Mass spectrometry

After immunoprecipitation, bound proteins were lysed in 8 M urea + 25 mM ammonium bicarbonate followed by reduction (5 mM dithiothreitol for 1 h at 37 °C), alkylation (10 mM iodoacetamide for 45 min at room temperature in the dark) and digestion overnight with 1 µg of trypsin (Promega). Peptide samples were applied to activated columns, and the columns were washed three times with 200 µl of 0.1% trifluoroacetic acid. Peptides were eluted with 140 µl of 50% acetonitrile and 0.1% trifluoroacetic acid and dried down by speedvac.

Samples were resuspended in 0.1% formic acid and separated by reversed-phase chromatography using an EASY-nLC instrument (Thermo Fisher Scientific) with a 15-cm PepSep column (inner diameter of 150 µm; Bruker). Samples were acquired by data-dependent acquisition. Mobile phase A consisted of 0.1% formic acid in water, and mobile phase B consisted of 80% acetonitrile and 0.1% formic acid. Peptides were separated at a flow rate of 500 nl min^−1^ over the following 60 min gradient: 4–35% B in 44 min, 35–45% B in 5 min and 10 min at 88% B. Peptides were analysed by an Orbitrap Lumos MS instrument (Thermo Fisher Scientific). Data were collected in positive ion mode with MS1 resolution of 240,000, 350–1,350 *m*/*z* scan range, maximum injection time of 50 ms, radiofrequency lens of 30%. For data-dependent acquisition, MS2 fragmentation was performed on charge states 2–5 with a 20-s dynamic exclusion after a single selection and 10 ppm ± mass tolerance. All raw mass spectrometry data were searched using MaxQuant (v2.4.7) against the human proteome (UniProt canonical protein sequences, downloaded in September 2022) using default settings and with a match-between-runs enabled^62^.

### Mass spectrometry analysis

Protein spectral counts as determined by MaxQuant search results were used for protein–protein interaction (PPI) confidence scoring by SAINTexpress^63^ (v3.6.1). Rabbit IgG pulldown samples were used as control. The total list of candidate PPIs was filtered to those that met the criteria of SAINTexpress Bayesian FDR ≤ 0.05. To quantify changes in interactions between resting and stimulated T cell states, we used a label-free quantification approach in which statistical analysis was performed using MSstats (v4.8.7)^64^ from the artMS (v1.18.0) R package. Visualization was performed in Cytoscape with additional connections included from the STRING database^65^.

### Western blots

After affinity purification of proteins, beads were resuspended in 100 µl 2X sample buffer (4× Laemmli Sample Buffer; 1610747, Bio-Rad) with 1:10 β-mercaptoethanol (63689-25ML-F, Sigma) diluted 1:1 with 500 µl lysis buffer. Samples were boiled for 5 min at 95 °C and stored at −20 °C until further processing. Western blots were performed as previously published^66^. In brief, cell lysates were subjected to SDS–PAGE on 4–15% acrylamide gels and electroblotted to polyvinylidene difluoride membranes. Blocking and primary (diluted 1:1,000) and secondary antibody incubations of immunoblots were performed in Tris-buffered saline + 0.1% Tween-20 supplemented with 5% (w/v) BSA (antibodies are provided in Supplementary Table 9). Horseradish peroxidase-conjugated goat anti-rabbit and IgG (Southern Biotech) were used at a dilution of 1:30,000, and immunoreactive bands were detected using Pierce ECL Western Blotting Substrate (32106) according to the manufacturer’s instructions.

### CUT&RUN

CUT&RUN was performed on resting and 48-h restimulated cells according to the manufacturer’s protocol with the EpiCypher CUTANA ChIC/CUT&RUN Kit and provided reagents. Samples for H3K27ac CUT&RUN were lightly crosslinked before isolation using 0.1% formaldehyde (252549, Sigma) for 1 min and quenched with 125 mM glycine (50046, Sigma). In brief, 5 × 10^5^ T cells per reaction were washed with PBS before nuclear isolation using the EpiCypher recommended lysis buffer consisting of 20 mM HEPES pH 7.9 (Sigma-Aldrich), 10 mM KCl (Sigma-Aldrich), 0.1% Triton X-100 (Sigma-Aldrich), 20% glycerol (Sigma-Aldrich), 1 mM MnCl_2_ (Sigma-Aldrich), 1X cOmplete Mini-Tablet (11873580001, Roche) and 0.5 mM spermidine (Sigma-Aldrich). The cells were resuspended in 100 µl per reaction cold nuclear extraction buffer and incubated on ice for 10 min. Following lysis, nuclei were pelleted and resuspended in 100 µl per reaction of nuclear extraction buffer. The isolated nuclei were then frozen at −80 °C in extraction buffer until DNA isolation. After thawing the samples at 37 °C, the nuclei were bound to activated conA beads. After adsorption of nuclei to beads, permeabilization was performed with 0.01% digitonin-containing buffer. Antibodies for H3K27ac (13-0045, EpiCypher), H3K4me1 (13-0057, EpiCypher), H3K4me2 (13-0027, EpiCypher), H3K4me3 (13-0041, EpiCypher) and IgG (13-0042, EpiCypher) were added at 500 ng per reaction. Following overnight antibody binding, pAG-MNase addition and chromatin cleavage, 0.5 ng of the provided *Escherichia coli* DNA was added to each sample following chromatin cleavage by MNase. Before DNA isolation, crosslinked samples were digested overnight with proteinase K (AM2546, Invitrogen) as recommended. The provided spin columns and buffers were used for DNA isolation and purification. The resulting DNA was prepared for sequencing using the CUTANA CUT&RUN Library Prep Kit (14-1002) according to the manufacturer’s protocol.

### CUT&RUN analysis

Pooled libraries were sequenced on a NextSeq 500 (H3K27ac) and NextSeq 2000 with 2 × 75 or 2 × 50 paired-end reads, respectively. Bcl2fastq (v2.19) with the settings --minimum-trimmed-read-length 8 was used to generate fastqs. CUT&RUN data analysis was performed according Zheng et al. with the recommended settings unless otherwise specified below^67^. In brief, the fastqs were trimmed with cutadapt (v1.18). Bowtie2 (v2.2.5)^68^ was used to align the trimmed fastqs to GRCh38 using settings --local --very-sensitive --no-mixed --no-discordant --phred33 --dovetail -I 10 -X 700 -p 8 -q and *E. coli* (EMBL accession U00096.2) with settings --local --very-sensitive --no-overlap --no-dovetail --no-mixed --no-discordant --phred33 -I 10 -X 700 -p 8 -q. Bam files were generated with SAMtools^69,70^ (v1.9) view -bS -F 0 × 04 and bam-to-bed conversion performed with bedtools (v2.30.0) bamtobed -bedpe. Bedfiles were filtered to include only paired reads of less than 1,000 bp with the command awk ‘$1==$4 & & $6-$2 < 1000 {print $0}’ samplename.bed before generating bedgraph files using bedtools (v2.30.0) genomecov -bg. Peak calling was performed using the bedgraph files as input with SEACR^71^ (v1.3). Each target bedgraph file was compared to the respective donor and knockout condition IgG file to identify peaks above the background using the norm and stringent options for H3K27ac samples. Spike-in scaling was performed before methylation peak calling with SEACR using the IgG file as background without normalization (non option) and with the stringent option.

Before generating a peak by sample matrix for each target, ChIP–seq blacklist regions were removed from the data. The sample matrix was reduced across all peaks within the dataset, and H3K27ac peaks were segmented into regions of 5,000 bp maximum length. Regions of differential acetylation or methylation between the regulator knockouts and *AAVS1*-knockout samples were identified for the peaks called across any of the samples from bam files using DESeq2 (v1.32.0)^51^ in R (v4.1.0; Supplementary Table 10). Comparisons were made within each cell-type and stimulation condition using AAVS1s prepared in the same batch of samples. Gene annotation was performed using the gene with the nearest TSS to each region with the GenomicRanges^72^ (v1.44.0) nearest function. Final bedgraph scaling was performed based on peak coverage across all samples and conditions using DESeq2 (v1.32.0) sizefactors. SEL120-34A and H_2_O treatment samples were compared as described for MED12-knockout and *AAVS1*-knockout samples, using the peak matrix from MED12-knockout and *AAVS1*-knockout samples to maximize detection of overlapping regions across datasets.

### ChIP–seq

A portion of edited T_eff_ cells were restimulated with ImmunoCult human CD3/CD28/CD2 T cell activator (10990, STEMCELL Technologies) 10 days following isolation and collected 48 h later. Up to 1–2 × 10^6^ T_eff_ cells were crosslinked in PBS with 1% methanol-free formaldehyde (28908, Thermo) for 10 min at 18–22 °C followed by quenching in glycine at 125 mM final concentration. Crosslinked cell pellets were snap-frozen in liquid nitrogen and stored at –80 °C. Nuclei were isolated from thawed, crosslinked cells via sequential lysis in LB1 (50 mM HEPES-KOH pH 7.5, 140 mM NaCl, 1 mM EDTA, 10% glycerol, 0.5% IGEPAL CA-360 and 0.25% Triton X-100), LB2 (10 mM Tris-HCl pH 8, 200 mM NaCl, 1 mM EDTA and 0.5 mM EGTA) and LB3 (10 mM Tris-HCl pH 8, 100 mM NaCl, 1 mM EDTA, 0.5 mM EGTA, 1% sodium deoxycholate (NaDOC) and 0.5% *N*-laurylsarcosine) supplemented with 0.5 mM phenylmethylsulfonyl fluoride (PMSF; P7626, Sigma) and 0.5X protease inhibitor cocktail (PIC; P8340, Sigma). Chromatin was sheared on a Covaris E220-focused ultrasonicator using 1-ml milliTubes (520128, Covaris) with 140 W peak incident power, 5% duty factor, 200 cycles per burst, 6 °C temperature setpoint (minimum of 3 °C and maximum of 9 °C), fill level 10, and time 12–14 min to obtain a target size of 200–700 bp. Formaldehyde crosslinked, sheared mouse CD8^+^ T cell chromatin was spiked in at 2.5% of human T_eff_ chromatin based on fluorometric (Qubit, Q33238, Thermo) or OD260 (Nanodrop, 912A1099, Thermo) quantification. Triton X-100 was added to a final concentration of 1% before immunoprecipitation for 16 h at 4 °C with 2–8 µg of indicated antibodies (Supplementary Table 9) bound to a 1:1 mixture of protein A and protein G magnetic beads (10001D and 10003D, Thermo). Bead-bound antibody–chromatin complexes were sequentially washed three times with wash buffer 1 (20 mM Tris pH 8, 150 mM NaCl, 1 mM EDTA, 0.5 mM EGTA, 1% Triton X-100, 0.1% SDS and 0.1% NaDOC), twice with wash buffer 2 (20 mM Tris-HCl pH 8, 500 mM NaCl, 1 mM EDTA, 0.5 mM EGTA, 1% Triton X-100, 0.1% SDS and 0.1% NaDOC), twice with wash buffer 3 (20 mM Tris-HCl pH 8, 250 mM LiCl, 1 mM EDTA, 0.5% IGEPAL CA-360 and 0.5% NaDOC), twice with TET (10 mM Tris-HCl pH 8, 1 mM EDTA and 0.2% Tween-20) and once with TE0.1 (10 mM Tris-HCl pH 8, 0.1 mM EDTA, 0.5 mM PMSF and 0.5X PIC) supplemented with 0.5 mM PMSF and 0.5X PIC. Beads were resuspended in TT (10 mM Tris-HCl pH 8 and 0.05% Tween-20) before on-bead library preparation using the NEBNext Ultra II DNA Library Prep Kit (E7370L, NEB) as previously described^73^. ChIP–seq libraries were multiplexed for paired-end (2 × 50 bp) sequencing on an Illumina NextSeq 2000 instrument.

### ChIP–seq analysis

Reads were trimmed to remove adapters and low-quality sequences and aligned to the hg38 and mm10 reference genome assemblies with bwa^74^ (v0.7.17-r1188) before filtering to remove duplicates and low-quality alignments including problematic genomic regions^75^ using the nf-core/ChIP–seq pipeline^76^ (v2.0.0; 10.5281/zenodo.3240506) with default parameters. Normalization to mouse spike-in chromatin was performed by scaling counts to the quotient of the ratios of human:mouse ChIP reads and human:mouse input reads as previously described^77^. CXXC1 peaks for visualization were identified using bam files from all *AAVS1*-knockout donors for MACS2 (v2.2.6)^78^ callpeak -q 0.05 with input samples used to define the background. High-confidence MED12 peaks were identified using bam files from all *AAVS1*-knockout donors for MACS2 callpeak -q 0.05 with MED12-knockout samples used to define the background (Supplementary Table 11). Utilization of high-confidence peaks generated from knockout controls reduced potential false-positive signals from the ChIP samples, providing a more rigorous assessment of MED12 binding^79,80^. ChIP–seq blacklist regions were removed from CXXC1 and MED12 peaks before analysis.

### Polymerase pausing analysis

The polymerase pausing index was calculated as previously described^33^ as (TSS coverage/TSS length)/(gene body coverage/gene body length). Gencode v43 gene structures were selected for APRIS genes and filtered to include only genes expressed in T_eff_ bulk RNA-seq data (defined from *AAVS1* T_eff_ RNA-seq base mean > 10). The TSS region of each gene was defined as 200 bp upstream and downstream of the TSS. The gene body was defined as the region 400 bp downstream from the TSS plus 400 bp past the final exon of the gene. Rtracklayer^81^ (v1.62.0) was used to import spike-in scaled RNA Pol II CTD bigwigs, and GenomicAlignments (v1.38.2) summarizeOverlaps() was used to determine the coverage within the defined gene regions.

### CUT&RUN and ChIP–seq visualization

Visualization of scaled tracks was performed with rtracklayer (v1.62.0) and ggplot2 (v3.5.1) with smoothing. APRIS gene structure was used for gene annotation with gggenes (v0.5.0). CD4^+^ T_reg_ STAT5A ChIP–seq data were accessed from ChIP Atlas^82^, SRX212432 and GSM1056923, and generated by Hoffmann et al.^31^. Deeptools (v3.5.5)^83^ was used to generate profile plots of ChIP–seq data using computeMatrix scale-regions -b 3000 --regionBodyLength 5000 -a 3000 –skipZeros with scaled bigwigs, and a bed file of all expressed genes (defined from *AAVS1* T_eff_ RNA-seq base mean > 10) as input, followed by plotProfile –perGroup.

### MED12 CAR activation scoring

MED12 CAR RNA-seq data from Freitas et al. was accessed from the Gene Expression Omnibus, using the downloader to retrieve the raw counts file (GSE174279_raw_counts_GRCh38.p13_NCBI.tsv.gz). First, DESeq2 (v1.32.0) was used to identify differentially expressed genes between *AAVS1*-knockout stimulated and resting samples. The top upregulated genes were defined using the following criteria: adjusted *P* < 0.01, log_2_ fold change > 2 and base mean > 10. The resulting 797 genes were used to generate a gene signature of activation. Normalized counts for the MED12-knockout and *AAVS1*-knockout resting and stimulated samples were generated with DESeq2 vst and converted to a summarized experiment with SummarizedExperiment^84^ (v1.22.0). The normalized count matrix and activation score were used as input for GSVA^85^ (v1.40.1) using the gsva function with min.sz=10, max.sz=6000, kcdf = ‘Poisson’. Visualization of the resulting gene scores was performed with ggplot2(v3.4.1) and adjusted *P* values were generated using rstatix (v0.7.2).

### Activation-induced cell death assays

Activation-induced cell death assays were performed using titrated amounts of ImmunoCult human CD3/CD28/CD2 T cell activator (10990, STEMCELL Technologies) in addition to 50 U ml^−1^ of IL-2. Active caspase-3/7 staining was performed 72 h following addition of stimulus using the CellEvent Caspase-3/7 Green Flow Cytometry Assay Kit (C10427, Invitrogen) according to the manufacturer’s protocol. Gating of the apoptotic population was performed on the lymphocyte gate and defined as active caspase-3/7 positive and SYTOX nucleic acid stain negative. FAS staining was performed using phycoerythrin anti-human CD95 (Fas) antibody (305608, BioLegend; diluted 1:50).

### Luminex assays

On day 12 following isolation for T_eff_ cells and day 8 following isolation for T_reg_ cells, cells were plated in 96-well plates in cytokine-free medium at a density of 2 × 10^5^ cells per well. Cells were restimulated with ImmunoCult human CD3/CD28/CD2 T cell activator (10990, STEMCELLl Technologies) and supernatant was collected after 24 h. The supernatant was stored at −80 °C until processing by EVE Technologies with the Luminex xMAP technology on the Luminex 200 system. After a serial titration to determine appropriate dilutions, samples were run in technical duplicate, and Luminex 48 plex human panel A was run for T_eff_ cells (diluted 1:20) and T_reg_ cells (diluted 1:5). The multi-species TGF 3 plex panel was also run for T_reg_ cells (undiluted). Technical replicates were averaged by EVE for each sgRNA and donor combination to determine protein concentration. Cytokines with more than one sample out of range were removed from the analysis to exclude low abundance proteins (Supplementary Table 12).

### Suppression assays

Donor-matched T_eff_ cells were isolated and frozen at −80 °C without activation until 24 h before the assay. T_eff_ cells were thawed and cultured overnight at 2 × 10^6^ cells per millilitre with 10 U ml^−1^ IL-2. On the day of the assay, T_eff_ cells were counted and stained with CellTrace Violet (C34557, Invitrogen) according to the manufacturer’s protocol using a 1:2,000 dilution of dye. Assay plates were assembled with 1 × 10^5^ T_eff_ cells per well in 96-well round bottom plates with titrated amounts of T_reg_ cells ranging from 1:1 to 8:1 T_eff_ cells:T_reg_ cells. One well per condition was also included of 1 × 10^5^ T_reg_ cells and 5 × 10^4^ T_eff_ cells (1:2 T_eff_ cells:T_reg_ cells), as well as resting and stimulated T_reg_ cells and T_eff_ cells individually as controls. T_reg_ Suppression Inspector (130-092-909, Miltenyi Biotec) iMACS particles were prepared and added to the appropriate wells according to the manufacturer’s recommendations. Assays were performed in technical triplicate for four donors, and plates were incubated for 96 h at 37 °C. At the time of readout, cells were stained with Alexa Fluor 647 anti-human IL-2Rα (302618, BioLegend), BV711 anti-human CD4 (344648, BioLegend) and Ghost Dye Red 780 (13-0865-T500, Tonbo), and analysed on the Attune NxT flow cytometer (A29004).

Analysis of flow data was performed in FlowJo (v10.8.1) with gating to select for lymphocytes, singlets, live cells (Ghost Dye negative), CD4^+^ T cells and T_eff_ cells (CellTrace Violet+CD25^low^). A gate was then set for each donor using the non-stimulated T_eff_-only control (CellTrace Violet high peak) to establish a proliferative T_eff_ count. A gate was also set for iMACS beads by selecting non-lymphocytes, beads using forward scatter area (FSC-A) and Ghost Dye. An absolute proliferating T_eff_ cell count was then established using the formula (proliferative T_eff_ cell count × input bead count)/(beads), which adjusts for variations in stimulation and collection abnormalities. Percentage suppression was calculated as (100 – (absolute proliferating T_eff_ cell count/absolute proliferating T_eff_ cell count of stimulated responder only condition)) × 100. The median of the technical replicate collection plates was used to calculate percent suppression and absolute proliferating T_eff_ cell count per donor for visualization.

### Reporting summary

Further information on research design is available in the Nature Portfolio Reporting Summary linked to this article.

## Online content

Any methods, additional references, Nature Portfolio reporting summaries, source data, extended data, supplementary information, acknowledgements, peer review information; details of author contributions and competing interests; and statements of data and code availability are available at 10.1038/s41586-024-08314-y.

## Supplementary information

Supplementary Fig. 1 Uncropped western blots relating to Extended Data Fig. 7c,d

Reporting Summary

Supplementary Tables Supplementary Tables 1–12

## Source data

Source Data Fig. 2

Source Data Fig. 3

Source Data Fig. 4

Source Data Fig. 5

Source Data Fig. 6

Source Data Extended Data Fig. 1

Source Data Extended Data Fig. 2

Source Data Extended Data Fig. 3

Source Data Extended Data Fig. 4

Source Data Extended Data Fig. 5

Source Data Extended Data Fig. 6

Source Data Extended Data Fig. 8

Source Data Extended Data Fig. 9

Source Data Extended Data Fig. 10

## Data Availability

IL-2Rα screens, CUT&RUN, ChIP–seq, bulk RNA-seq and Perturb–CITE-seq data are accessible at the NCBI Gene Expression Omnibus (GEO) within GEO SuperSeries GSE271090. Mass spectrometry proteomics data have been deposited to the ProteomeXchange Consortium via the PRIDE partner repository with the dataset identifier PXD056255. Publicly available data used in this study are accessible from the following sources: KLF2-knockout differentially expressed genes are available in Freimer et al.^19^. MED12 CAR-T bulk RNA-seq data are available in the GEO: GSE174279. CD4^+^ T_reg_ STAT5A ChIP–seq data are available in the ChIP Atlas with the identifiers SRX212432 and GSM105692. Source data are provided with this paper.

## Code availability

The code for main figure generation and key analyses is available on Zenodo^86^ (10.5281/zenodo.13924126).
