# Supplementary material for: Central control of dynamic gene circuits governs T cell rest and activation
Source: Nature. 2024 Dec 11;637(8047):930–9. doi: 10.1038/s41586-024-08314-y (PMC11754113; doi:10.1038/s41586-024-08314-y)

nature portfolio

https://doi.org/10.1038/s41586-024-08314-y

Supplementary information

Central control of dynamic gene circuits governs T cell rest and activation

In the format provided by the authors and unedited

Related to Extended Data Figure 7c,
N=3 replicates, representative blots shown below

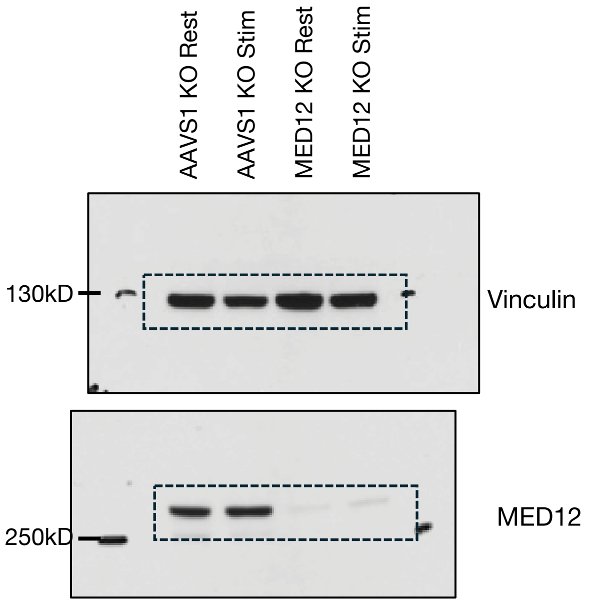

Related to Extended Data Figure 7d,
N=3 replicates, representative blots shown below

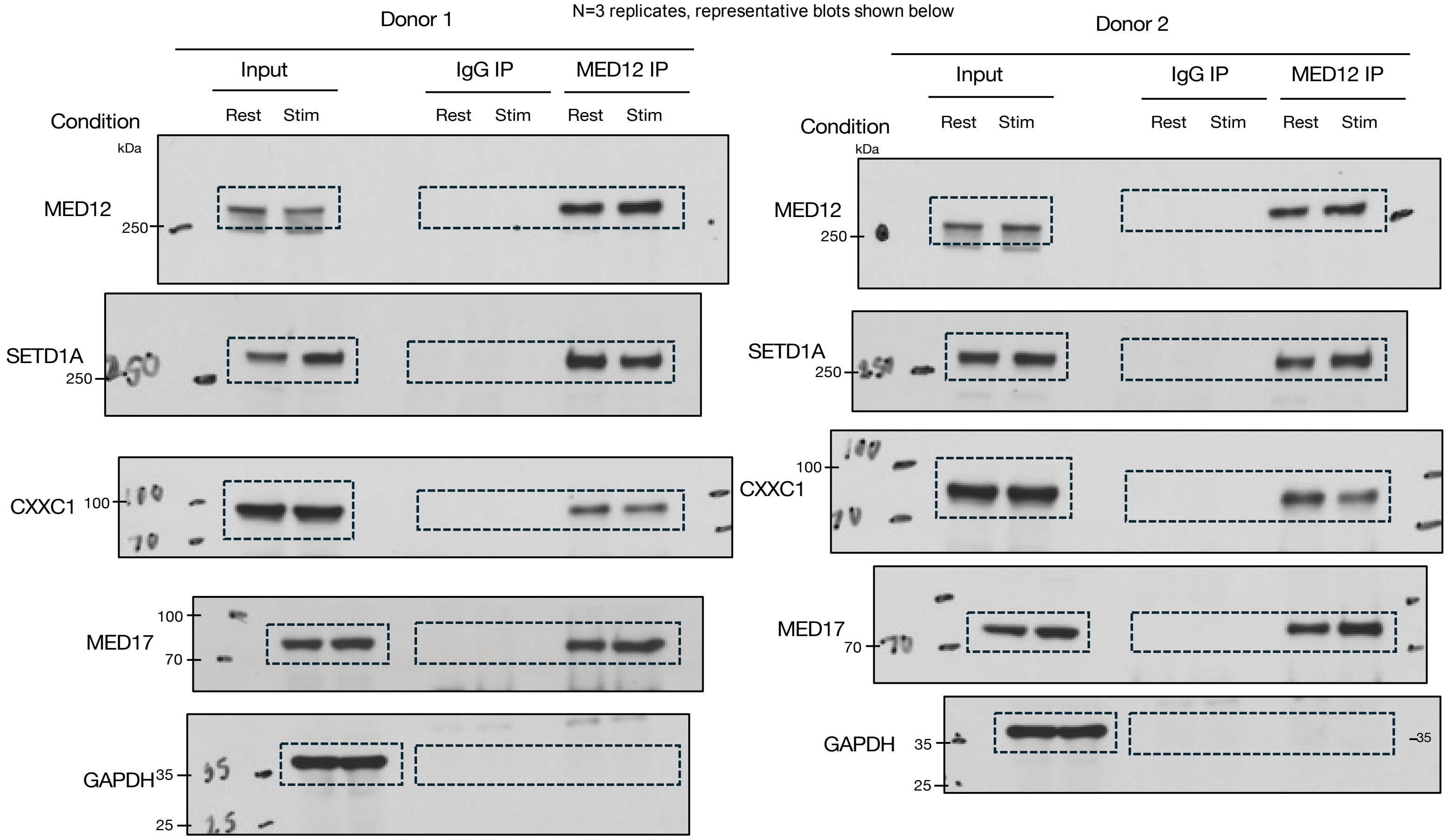

Supplement: Supplementary file 1 — Uncropped western blots relating to Extended Data Fig. 7c,d [file 41586_2024_8314_MOESM1_ESM.pdf]
