# Supplementary material for: Central control of dynamic gene circuits governs T cell rest and activation
Source: Nature. 2024 Dec 11;637(8047):930–9. doi: 10.1038/s41586-024-08314-y (PMC11754113; doi:10.1038/s41586-024-08314-y)
Supplement: Supplementary file 2 — Reporting Summary [file 41586_2024_8314_MOESM2_ESM.pdf]

nature portfolio

Corresponding author(s): Alexander Marson

Last updated by author(s): Oct 11, 2024

Reporting Summary

Nature Portfolio wishes to improve the reproducibility of the work that we publish. This form provides structure for consistency and transparency in reporting. For further information on Nature Portfolio policies, see our Editorial Policies and the Editorial Policy Checklist.

Statistics

For all statistical analyses, confirm that the following items are present in the figure legend, table legend, main text, or Methods section.

n/a	Confirmed	
☐	☒	The exact sample size (*n*) for each experimental group/condition, given as a discrete number and unit of measurement
☐	☒	A statement on whether measurements were taken from distinct samples or whether the same sample was measured repeatedly
☐	☒	The statistical test(s) used AND whether they are one- or two-sided<br>*Only common tests should be described solely by name; describe more complex techniques in the Methods section.*
☐	☒	A description of all covariates tested
☐	☒	A description of any assumptions or corrections, such as tests of normality and adjustment for multiple comparisons
☐	☒	A full description of the statistical parameters including central tendency (e.g. means) or other basic estimates (e.g. regression coefficient) AND variation (e.g. standard deviation) or associated estimates of uncertainty (e.g. confidence intervals)
☐	☒	For null hypothesis testing, the test statistic (e.g. *F*, *t*, *r*) with confidence intervals, effect sizes, degrees of freedom and *P* value noted<br>*Give P values as exact values whenever suitable.*
☒	☐	For Bayesian analysis, information on the choice of priors and Markov chain Monte Carlo settings
☒	☐	For hierarchical and complex designs, identification of the appropriate level for tests and full reporting of outcomes
☒	☐	Estimates of effect sizes (e.g. Cohen's *d*, Pearson's *r*), indicating how they were calculated

Our web collection on statistics for biologists contains articles on many of the points above.

Software and code

Policy information about availability of computer code

Data collection	Cell sorter data collection was performed with BD FACSDiva v9.0.1. Attune Cytometric software (v6.0.1) was used for data collection. RNAseq data was collected on an Illumina NextSeq 500, CUT&RUN and ChIPseq data were collected on an Illumina NextSeq 500 and NextSeq 2000, Perturb-seq was collected on an Illumina NovaSeq X.
Data analysis	Screen analysis<br>All pooled screens were analyzed with MAGeCK (v0.5.9.5).<br><br>RNAseq data was processed using the pipeline described in Freimer et al. Fastq adapter trimming was performed with cutadapt (v2.10). Low-quality bases were trimmed with seqtk (v0.5.0). Reads were then aligned with STAR(v 2.7.10a) and mapped to GRCh38. UMI counting and deduplication was performed with umi_tools (v1.0.1) and gene counts were generated from the deduplicated reads using featureCounts (subread v2.0.1). Quality control metrics were generated for each sample with Fastqc (v0.11.9), rseqc (v3.0.1), and Multiqc (v1.9). Differentially expressed genes between Mediator KOs and AAVS1 KO samples as well as stimulated and resting AAVS1 KO samples were identified from the deduplicated count matrix using Deseq247 (v1.32.0) in R (v4.1.0) Pathway analysis was performed using PathfindR (v1.6.4). Pathway visualization was performed using Cytoscape (v3.8.2).<br><br>CUT&RUN data analysis was performed according Zheng et al. with the recommended settings unless otherwise specified below62. In brief, the fastqs were trimmed with cutadapt (v1.18). Bowtie2 (v2.2.5) was used to align the trimmed fastqs. Bam files were generated with samtools(version 1.9) and bam to bed conversion performed with bedtools (v2.30.0). Bedgraph files were generated with bedtools (version 2.30.0). Peak calling was performed using the bedgraph files as input with SEACR (v1.3). Regions of differential acetylation or methylation between the regulator KOs and AAVS1 KO samples were identified for the peaks called across any of the samples from bam files using Deseq261 (v1.32.0) in R (v4.1.0). Gene annotation was performed using the gene with the nearest transcription start site to each region with

the GenomicRanges (v1.44.0) nearest function. Bedgraph scaling was performed based on peak coverage across all samples and conditions using Deseq2 (v1.32.0) sizefactors.

ChIP-seq analysis
Reads were trimmed to remove adapters and low-quality sequences and aligned with bwa59 (v0.7.17-r1188) before filtering to remove duplicates and low-quality alignments including problematic genomic regions60 using the nf-core/ChIP-seq pipeline61 (v2.0.0, doi: 10.5281/zenodo.3240506) with default parameters. Normalization to mouse spike-in chromatin was performed by scaling counts to the quotient of the ratios of human:mouse ChIP reads and human:mouse input reads as described62. CXXC1 and MED12 high confidence peaks for visualization were identified using bam files from all AAVS1 KO donors for MACS2 (v2.2.6).

Polymerase pausing analysis
The polymerase pausing index was calculated as described in Wang et al. as (TSS coverage/TSS length)/(Gene body coverage/gene body length).34 Gencode V43 gene structures were selected for APRIS genes and filtered to include only genes expressed in CD4+ T cells using AAVS1 KO bulk RNAseq data. The TSS region of each gene was defined as 200 bp up- and downstream of the TSS. The gene body was defined as the region 400 bp downstream from the TSS plus 400 bp past the final exon of the gene. Rtracklayer (v1.62.0) was used to import spike-in scaled RNA Pol II CTD bigwigs and GenomicAlignments (v1.38.2) summarizeOverlaps() was used to determine the coverage within the defined gene regions.

CUT&RUN and ChIP-seq visualization
Visualization of scaled tracks was performed with rtracklayer (v1.62.0) and ggplot2 (v3.5.1). Gene annotation was performed with gggenes (v0.5.0). Deeptools (v3.5.5) was used to generate profile plots.

Perturb-seq analysis
Perturb-seq analysis was performed in R (v4.3.1) using Seurat (v4.3.0.1).Harmony (v0.1.1) was used to correct for donor associated variability. DeSeq2 (v1.32.0) was used to identify differentially expressed genes and proteins. Network plots of differentially expressed gene connections were visualized in R using influential (v2.2.7) and ggraph (v2.1.0). Other visualization of differentially expressed genes and surface proteins was performed using ggplot2 (v3.4.1). Activation scoring was performed according to Schmidt et at 2022.

Mass Spectrometry analysis
All raw MS data were searched using MaxQuant (v2.4.7). Protein spectral counts as determined by MaxQuant search results were used for PPI confidence scoring by SAINTexpress (v3.6.1). Rabbit IgG pulldown samples were used as control. The total list of candidate PPIs was filtered to those that met the criteria of SAINTexpress BFDR <= 0.05. To quantify changes in interactions between resting and stimulated T cell states, we used a label-free quantification approach in which statistical analysis was performed using MSstats (v4.8.7) within the artMS (v1.18.0) R package.

MED12 CAR activation scoring
DeSeq2 (v1.32.0), SummarizedExperiment (v1.22.0), GSVA68 (v1.40.1) and rstatix (v0.7.2) were used.

For manuscripts utilizing custom algorithms or software that are central to the research but not yet described in published literature, software must be made available to editors and reviewers. We strongly encourage code deposition in a community repository (e.g. GitHub). See the Nature Portfolio guidelines for submitting code & software for further information.

Data

Policy information about availability of data

All manuscripts must include a data availability statement. This statement should provide the following information, where applicable:

- Accession codes, unique identifiers, or web links for publicly available datasets
- A description of any restrictions on data availability
- For clinical datasets or third party data, please ensure that the statement adheres to our policy

IL2RA screens, CUT&RUN, ChIP-seq, Bulk RNAseq, and Perturb-CITE-seq data are accessible at NCBI Gene Expression Omnibus within GEO SuperSeries GSE271090. Mass spectrometry proteomics data have been deposited to the ProteomeXchange Consortium via the PRIDE partner repository with the dataset identifier PXD056255. Source data has been provided for figures where the data is not accessible from the supplementary tables. MED12 CAR-T bulk RNAseq data is available on GEO: GSE174279. CD4+ Treg STAT5A ChIP-seq data was accessed from ChIP Atlas- SRX212432, GSM105692.

Research involving human participants, their data, or biological material

Policy information about studies with human participants or human data. See also policy information about sex, gender (identity/presentation), and sexual orientation and race, ethnicity and racism.

Reporting on sex and gender	Donors were recruited by StemCell technologies and were used in this study without regard to sex or gender.
Reporting on race, ethnicity, or other socially relevant groupings	Donors were recruited by StemCell technologies and were used in this study without regard to race, ethnicity, or other social groupings.
Population characteristics	For experiments with large quantities of regulatory T cells, donors were requested under the age of 40 with a BMI of less than 30 to improve regulatory T cell yield. Beyond this criteria, blood was accepted as available from STEMCELL Technologies without other selection criteria. STEMCELL provides the following information, "Subjects are voluntary, healthy donors, recruited from the general community, and compensated for their time and effort. Criteria for entry into the program includes age (minimum: 18 years old; maximum: 55 years old or higher, depending on the donor site), and donors without

	any pre-existing conditions such as cancer, cardiac, lung, blood, or autoimmune disorders. Subjects are recruited without regard for ethnicity, although US citizenship is required."
Recruitment	All samples were purchased through STEMCELL Technologies who was soley responsible for recruitment of donors. STEMCELL Technologies collects blood from healthy human donors.
Ethics oversight	All biospecimens were purchased through STEMCELL Technologies which collects after institutional review board–approved informed written consent.

Note that full information on the approval of the study protocol must also be provided in the manuscript.

Field-specific reporting

Please select the one below that is the best fit for your research. If you are not sure, read the appropriate sections before making your selection.

☒ Life sciences ☐ Behavioural & social sciences ☐ Ecological, evolutionary & environmental sciences

For a reference copy of the document with all sections, see nature.com/documents/nr-reporting-summary-flat.pdf

Life sciences study design

All studies must disclose on these points even when the disclosure is negative.

Sample size	Sample sizes were selected based on standards for the specific data type and downstream analysis pipelines. Human biological replicate samples were used in all experiments and in some cases multiple CRISPR guides targeting the same gene. We determined sample sizes based on prior experiments performed in our labs and published findings of the same data type. The chosen sample sizes reproducibly yielded reported effects.
Data exclusions	For screens, donors with insufficient cell yield after isolation, cell culturing, or sorting were excluded from the analysis. Only donors with over 500x coverage were included in the dataset to ensure appropriate data quality (resulting in the exclusion of 1 Treg donor with poor expansion). In arrayed flow cytometry experiments, samples with insufficient total events (ie less than 1000 total cells per well) were removed from the analysis using a consistent threshold across the entire dataset. Samples with insufficient reads at the edited locus were excluded from genotyping analysis with a consistent threshold used across the entire dataset (ie less than 100 reads). A pilot experiment donor was processed for ChIP-seq but excluded due to poor signal to noise ratio and the use of non-optimal antibodies. ChIP-seq included in the manuscript was carried out after replacing sub-optimal antibodies and improving the stringency of washes.
Replication	Human biological replicates were used in all experiments to ensure reproducibility. For most experiments, cells from multiple donors were cultured in parallel in batches of 1-4 donors at a time. For several large arrayed assays, downstream processing of each donor was performed separately for each donor. Reproducibility across donors and batches of donors was high. Many of the findings in this work were validated or reproduced with a secondary method. Specifically, all featured IL2RA screen hits were assessed in an arrayed KO format with flow cytometry using independent donors. Mediator RNAseq analysis was performed with CRISPR KO in an arrayed format and CRISPRi scRNAseq in individual donors. Conclusions were drawn from results observed using both CRISPR and sequencing modalities. CUT&RUN data peak calling was performed using the most stringent setting of SEACR, including IgG controls for background thresholding generated from each sample. Activation induced cell death flow data was generated in two separate experiments with two donors and two guides per gene in each experiment. All attempts at replication were successful and are included in the figures. ChIPseq peak calling was performed using the input as background for each sample and MED12 KO samples for MED12 peaks in AAVS1 KO control samples. IgG controls were used for all IP-MS and IP-western blot experiments.
Randomization	CRISPR perturbations performed in an arrayed format were randomized prior to the start of each experiment, including the exclusion of edge wells whenever possible. High throughput genomic assays that required batch processing were conduced with control samples evenly distributed within each batch to prevent inconsistencies. Each donor sample was included in the experimental and control groups of each assay performed. Several assays were performed in a pooled setting to eliminate potential variability, including all screens and CRISPRi scRNA-seq/ CITE-seq.
Blinding	Blinding was not possible as the individual who designed the experiments was often involved in conducting the experiment. To avoid biases, distribution of control and experiment conditions were integrated as well as possible for arrayed assays (example: each row of CRISPR KOs contains an AAVS1 KO control, or AAVS1 KO controls are present in plate every 20 wells) prior to the start of the experiment and multichannel pipetting techniques were used whenever possible to ensure equal treatment of all samples. For large scale culture experiments not in a plate, cells were cultured in parallel for each donor with same media and split at equal densities at the same frequency.

Reporting for specific materials, systems and methods

We require information from authors about some types of materials, experimental systems and methods used in many studies. Here, indicate whether each material, system or method listed is relevant to your study. If you are not sure if a list item applies to your research, read the appropriate section before selecting a response.

Materials & experimental systems

n/a	Involved in the study
☐	☒ Antibodies
☒	☐ Eukaryotic cell lines
☒	☐ Palaeontology and archaeology
☒	☐ Animals and other organisms
☒	☐ Clinical data
☒	☐ Dual use research of concern
☒	☐ Plants

Methods

n/a	Involved in the study
☐	☒ ChIP-seq
☐	☒ Flow cytometry
☒	☐ MRI-based neuroimaging

Antibodies

Antibodies used

Target Species/Isotype/Clone Vendor Cat. No.
MED12 Rabbit polyclonal IgG Bethyl/Thermo A300-774A
CXXC1 Rabbit monoclonal IgG (D1R5R) Cell Signaling 40672S
NELF-A Mouse monoclonal IgG2bk (G-11) Santa Cruz sc-365004
RNA PolII CTD Mouse monoclonal IgG1 (4H8) Cell Signaling 2629
RNA PolII phospho-Ser2 Rabbit polyclonal IgG Abcam ab5095
RNA PolII phosphor-Ser5 Rabbit polyclonal IgG Abcam ab5131
H3K27ac Rabbit Monoclonal (2114-3E4) EpiCypher 13-0045
H3K4me1 Rabbit Monoclonal (2088-1F4) EpiCypher 13-0057
H3K4me2 Rabbit Monoclonal (clone not provided by manufacturer website) EpiCypher 13-0027
H3K4me3 Rabbit Monoclonal (2909-3D7) EpiCypher 13-0041
IgG Rabbit EpiCypher 13-0042
MED12 Rabbit monoclonal IgG (D9K5J) Cell Signaling 14360
IgG Normal Rabbit IgG Cell Signaling 2729S
MED12 Rabbit monoclonal IgG (D9K5J) Cell Signaling 14360
IgG-HRP conjugate Mouse Anti-rabbit IgG (Conformation Specific) (L27A9) Cell Signaling 5127S
CXXC1 Rabbit monoclonal IgG (D1R5R) Cell Signaling 40672S
SET1A Rabbit mAb (D3V9S) Cell Signaling 61702
MED17 Rabbit mAb (E3V6Y) Cell Signaling 64733S
GAPDH Rabbit mAb D16H11 Cell Signaling 5174
Alexa Fluor® 647 anti-human IL2RA Mouse IgG1, κ (BC96) Biolegend 302618
Ghost Dye™ Red 780 NA Tonobo 13-0865-T500
BV711 anti-human CD4 Mouse IgG1, κ (SK3) Biolegend 344648
PE anti-mouse/human Helios Armenian Hamster IgG, 22F6 Biolegend 137216
KIRAVIA Blue 520™ anti-human CD152 (CTLA-4) Mouse IgG1, κ (L3D10) Biolegend 349938
Pacific Blue™ anti-human FOXP3 Mouse IgG1, κ (206D) Biolegend 320116
PE anti-Human CD127 Mouse IgG1, κ (HIL-7R-M21) Beckon Dickinson 557938
Pacific Blue™ anti-human CD4 Mouse IgG1, κ (SK3) Biolegend 344620
PE anti-human CD95 (Fas) Mouse IgG1, κ (DX2) Biolegend 305608
TotalSeq™-C0251 anti-human Hashtag Antibodies 1-4 Mouse IgG1 (LNH-94; 2M2) Biolegend 394661

Validation

All flow cytometry antibodies were validated and quality tested on the manufacturer website including a histogram of positive and negative cells stained with the respective product. Prior to using each antibody, we performed testing on the cell types of interest using relevant fixation methods to determine specificity and robust detection without significant spillover into the channels of other markers used in the study.

Biolegend antibody statement (manufacturer of most flow antibodies used) At BioLegend, one way that we address reproducibility in research is through antibody validation. We guarantee antibody specificity of all of our antibody products.

Manufacturer validation flow antibodies:
Alexa Fluor® 647 anti-human IL2RA Mouse IgG1, κ (BC96) Biolegend 302618 NA Flow cytometry https://www.biolegend.com/de-at/products/alexa-fluor-647-anti-human-cd25-antibody-3254
Ghost Dye™ Red 780 NA Tonobo 13-0865-T500 NA Flow cytometry
BV711 anti-human CD4 Mouse IgG1, κ (SK3) Biolegend 344648 NA Flow cytometry https://www.biolegend.com/fr-ch/products/brilliant-violet-711-anti-human-cd4-antibody-16013?GroupID=GROUP28
PE anti-mouse/human Helios Armenian Hamster IgG, 22F6 Biolegend 137216 NA Flow cytometry https://www.biolegend.com/ja-jp/products/pe-anti-mouse-human-helios-antibody-6481
KIRAVIA Blue 520™ anti-human CD152 (CTLA-4) Mouse IgG1, κ (L3D10) Biolegend 349938 NA Flow cytometry https://www.biolegend.com/fr-fr/products/kiravia-blue-520-anti-human-cd152-ctla-4-antibody-20315
Pacific Blue™ anti-human FOXP3 Mouse IgG1, κ (206D) Biolegend 320116 NA Flow cytometry https://www.biolegend.com/de-at/products/pacific-blue-anti-human-foxp3-antibody-3053
PE anti-Human CD127 Mouse IgG1, κ (HIL-7R-M21) Beckon Dickinson 557938 NA Flow cytometry https://www.bdbiosciences.com/en-us/products/reagents/flow-cytometry-reagents/research-reagents/single-color-antibodies-ruo/pe-mouse-anti-human-cd127.557938
Pacific Blue™ anti-human CD4 Mouse IgG1, κ (SK3) Biolegend 344620 NA Flow cytometry https://www.biolegend.com/en-gb/products/pacific-blue-anti-human-cd4-antibody-6507
PE anti-human CD95 (Fas) Mouse IgG1, κ (DX2) Biolegend 305608 NA Flow cytometry https://www.biolegend.com/en-gb/products/pe-anti-human-cd95-fas-antibody-643

CUT&RUN antibodies were certified by Epicypher and in the case of IgG provided as a control within the commercial kit used for sample processing. All CUT&RUN antibodies were tested in primary human T cells as recommended by EpiCypher prior to generation of data included in this manuscript.
EpiCypher's statement of quality:
- H3K427ac "This antibody meets EpiCypher's "SNAP-ChIP® Certified" criteria for specificity and efficient target enrichment in a ChIP experiment (<20% cross-reactivity across the panel, >5% recovery of target input). Histone H3 is one of the four proteins that are present in the nucleosome, the basic repeating subunit of chromatin, consisting of 147 base pairs of DNA wrapped around an octamer of core histone proteins (H2A, H2B, H3 and H4). This antibody reacts to H3K27ac and no cross reactivity with other lysine acylations in the EpiCypher SNAP-ChIP K-AcylStat panel, is detected."
- H3K4me3 (histone H3 lysine 4 trimethyl) antibody meets EpiCypher's lot-specific SNAP-Certified™ criteria for specificity and efficient target enrichment in CUT&RUN. This requires <20% cross-reactivity to related histone PTMs determined using the SNAP-CUTANA™ K-MetStat Panel of spike-in controls (EpiCypher 19-1002, Figure 1). High target efficiency is confirmed by consistent genomic enrichment at 500k and 50k starting cells (Figures 2-4). This antibody targets histone H3 trimethylated at lysine 4, which is enriched at active promoters near transcription start sites (TSS).
- H3K4me2:This antibody meets EpiCypher's lot-specific SNAP-Certified™ criteria for specificity and efficient target enrichment in CUT&RUN. This requires <20% cross-reactivity to related histone PTMs determined using the SNAP-CUTANA™ K-MetStat Panel of spike-in controls (EpiCypher 19-1002, Figure 1). High target efficiency is confirmed by consistent genomic enrichment at 500k and 50k starting cells (Figures 2-4). This antibody targets histone H3 dimethylation at lysine 4, which is enriched in promoters of transcriptionally active genes and genes primed for expression during cell development [1].
- H3K4me1 (histone H3 lysine 4 monomethyl) antibody meets EpiCypher's lot-specific SNAP-Certified™ criteria for specificity and efficient target enrichment in both CUT&RUN and CUT&Tag applications. This requires <20% cross-reactivity to related histone PTMs determined using the SNAP-CUTANA™ K-MetStat Panel of spike-in controls (EpiCypher 19-1002, Figures 1 and 4). High target efficiency is confirmed by consistent genomic enrichment at varying cell inputs: 500k and 50k cells in CUT&RUN (Figures 2-3); 100k and 10k cells in CUT&Tag (Figures 5-6). High efficiency antibodies display similar peak structures at representative loci (Figures 3 and 6) and highly conserved genome-wide signal (Figures 2 and 5) even at reduced cell numbers. H3K4me1 either flanks H3K4me3 at the transcription start site (TSS) or coincides with H3K4me3 (Figures 2-3, 5-6) [1].

Antibodies used for IP and western blot were validated for the respective assay by the manufacturer. The following statements detail validation strategies utilized by the relevant manufacturers.

Cell Signaling Antibody Validation for Western Blotting
Cell Signaling Technology (CST) provides the highest quality primary and secondary antibodies available for western blotting. CST™ antibodies are produced in-house and validated extensively according to a rigorous protocol.
Validation Steps Include
Examination of several cell lines and/or tissues of known expression levels allows accurate determination of species cross-reactivity and verifies specificity.
Treatment of cell lines with growth factors, chemical activators or inhibitors, which induce or inhibit target expression, verifies specificity. Phosphatase treatment confirms phospho-specificity.
The use of siRNA transfection or knockout cell lines verifies target specificity.
Side-by-side comparison of lots to ensures lot-to-lot consistency.
Optimal dilutions and buffers are predetermined, positive and negative cell extracts are specified, and detailed protocols are already optimized, saving valuable time and reagents.

We performed secondary validation of the antibody used for MED12 IPs using western blot after CRIPSR KO of MED12. We found that in our hands the antibody was both sensitive and specific for MED12. (Extended data figure).

Manufacturer validation:
MED12 Rabbit monoclonal IgG (D9K5J) Cell Signaling 14360 10 IP https://www.cellsignal.com/products/primary-antibodies/med12-d9k5j-rabbit-mab/14360?srsltid=AfmBOoqk6__foBrMyq1Gt4Er80ayxQJp0MtfSAM3trtTGTg70Asgsecd
IgG Normal Rabbit IgG Cell Signaling 2729S 10 IP https://www.cellsignal.com/products/primary-antibodies/normal-rabbit-igg/2729
MED12 Rabbit monoclonal IgG (D9K5J) Cell Signaling 14360 NA Western blot https://www.cellsignal.com/products/primary-antibodies/med12-d9k5j-rabbit-mab/14360?srsltid=AfmBOoqk6__foBrMyq1Gt4Er80ayxQJp0MtfSAM3trtTGTg70Asgsecd
IgG-HRP conjugate Mouse Anti-rabbit IgG (Conformation Specific) (L27A9) Cell Signaling 5127S NA Western blot https://www.cellsignal.com/products/secondary-antibodies/mouse-anti-rabbit-igg-conformation-specific-l27a9-mab-hrp-conjugate/5127?srsltid=AfmBOoqlM4WDa0H1xDhjGVUk4F9j7da0gbRCIVxXCoi8vX-Mz8fd4ZnA
CXXC1 Rabbit monoclonal IgG (D1R5R) Cell Signaling 40672S NA Western blot https://www.cellsignal.com/products/primary-antibodies/cxxc1-d1r5r-rabbit-mab/40672?srsltid=AfmBOopbUWhOQpRmNLLuqTS3FzsprF7r0aNPYwhYexWWZ6goIhmbJgph
MED17 Rabbit mAb (E3V6Y) Cell Signaling 64733S NA Western blot https://www.cellsignal.com/products/primary-antibodies/med17-e3v6y-rabbit-mab/64733?srsltid=AfmBOoqEtvuLb6Q79sMY_Rr7IAUqfM8junXdNkZOAskKrvOwNHkQvNK5
SET1A Rabbit mAb (D3V9S) Cell Signaling 61702 NA Western blot https://www.cellsignal.com/products/primary-antibodies/set1a-d3v9s-rabbit-mab/61702?srsltid=AfmBOorAh-0wsg-rUpoHiSAmgkq4v7qkVmdUbaLxyjoUBPDQkJrKbJdq
GAPDH Rabbit mAb D16H11 Cell Signaling 5174 NA Western blot https://www.cellsignal.com/products/primary-antibodies/gapdh-d16h11-xp-rabbit-mab/5174?srsltid=AfmBOoo-z5k8jbSK_ITrD0LRomimur8oB5Ic1oQ6LlMV2QKvByQ-SqLe

Cell signaling and Abcam ChIP-seq antibodies were validated by the manufacturer. The following statement is provided regarded manufacturer validation of antibodies.

Cell Signaling ChIP-seq Antibody Validation Steps
All ChIP-seq validated antibodies are first subjected to the ChIP-qPCR validation protocol.
Antibody sensitivity for ChIP-seq is then confirmed by analyzing the signal:noise ratio of target enrichment across the genome in antibody:input control comparisons. The antibody must provide an acceptable minimum number of defined enrichment peaks and a minimum signal:noise threshold compared to input chromatin.
For sequence-specific DNA-binding transcription factors, antibody specificity is determined by performing motif analysis of enriched chromatin fragments.
Antibody specificity is further determined by comparing enrichment across the genome using multiple antibodies against distinct

target protein epitopes.
Antibody specificity is confirmed using antibodies against different subunits of a multiprotein complex.
Antibody specificity is further confirmed by comparing enrichment across the genome to published ChIP-seq data (ie, ENCODE) using additional antibodies for a given target protein.

Relevant citation for the products include:
CD4 SK3 clone:
Garcia–Perez JE, et al. 2019. Front Immunol. 10:998. PubMed
Chiu Y, et al. 2016. Sci Rep. 6:19227. PubMed
Kuo HH, et al. 2018. Immunity. 48:1183. PubMed
Kariminia A, et al. 2016. Blood. 127: 3082 - 3091. PubMed

CD25 BC96 clone:
Lin JR et al. 2018. eLife. 7 pii: e31657. PubMed
Kilpelainen A, et al. 2022. Front Immunol. 13:815041. PubMed
Tiittanen M, et al. 2013. PLoS One. 7:78420. PubMed
Singh KS, et al. 2021. Nature. 589:597. PubMed
Keck S, et al. 2021. Cellular and Molecular Gastroenterology and Hepatology. 12(2):507-545. PubMed

Helios 22F6 clone:
Nagai Y, et al. 2019. Front Immunol. 10:174. PubMed
Sasaki K, et al. 2019. Nat Commun. 10:3878. PubMed
Dean JW, et al. 2020. J Autoimmun. 108:102417. PubMed
Baine I, et al. 2013. J Immunol. 190:1008. PubMed
Trotta E, et al. 2018. Nat Med. 24:1005. PubMed

FoxP3 206D clone:
Raghavan S, et al. 2009. Ann Rheum Dis. 68:1908. PubMed
Purvis H, et al. 2010. Blood. 116:4829. PubMed
Hartigan-O'Connor D, et al. 2007. J Exp Med. 204:2679. PubMed
Harshe RP, et al. 2020. Nat Commun. 11:5894. PubMed

MED12 Rabbit monoclonal IgG (D9K5J) Cell Signaling 14360- Western blot and IP
https://www.cellsignal.com/products/primary-antibodies/med12-d9k5j-rabbit-mab/14360?srsltid=AfmBOoqV3X0w8_caN70wpaCb5x8S9PLtIw4leAHf_NI_XHcpfxTGrfD3
Chen H, et al. Mol Cancer Ther. 2022 Jul 5;21(7):1076-1089. doi: 10.1158/1535-7163.MCT-21-0841. PMID: 35439318; PMCID: PMC9769698.
Siraj AK, et al. MED12 is recurrently mutated in Middle Eastern colorectal cancer. Gut. 2018 Apr;67(4):663-671. doi: 10.1136/gutjnl-2016-313334. Epub 2017 Feb 9. PMID: 28183795; PMCID: PMC5868237.

H3K27ac- CUT&RUN (Epicypher)
Shah et al., Mol Cell 2018

MED12 Rabbit polyclonal IgG Bethyl/Thermo A300-774A - ChIPseq
https://www.fortislife.com/products/primary-antibodies/rabbit-anti-med12-antibody/BETHYL-A300-774
Freitas KA, et al. Science. 2022 Nov 11;378(6620):eabn5647. doi: 10.1126/science.abn5647. Epub 2022 Nov 11. PMID: 36356142; PMCID: PMC10335827.
Sooraj D, et al. Mol Cell. 2022 Jan 6;82(1):123-139.e7. doi: 10.1016/j.molcel.2021.11.015. Epub 2021 Dec 14. PMID: 34910943.

CXXC1 Rabbit monoclonal IgG (D1R5R) Cell Signaling 40672S
https://www.cellsignal.com/products/primary-antibodies/cxxc1-d1r5r-rabbit-mab/40672?srsltid=AfmBOoq09oPFx92lhzjmhpiRTqjLEKN3w-rtr9xknfptKq1fYMDh3Bj8
Ding Y, et al. PLoS Pathog. 2021 Sep 7;17(9):e1009847. doi: 10.1371/journal.ppat.1009847. PMID: 34492084; PMCID: PMC8448337.
Zhang Q, et al. 2020 Jan-Dec;19:1533033820971306. doi: 10.1177/1533033820971306. PMID: 33174521; PMCID: PMC7672768.

SET1A Rabbit mAb (D3V9S) Cell Signaling 61702
https://www.cellsignal.com/products/primary-antibodies/set1a-d3v9s-rabbit-mab/61702?srsltid=AfmBOopMvZZBAqJhgNClSyv4vABVzcekCTbuGYWGZ1VKCcxRfWmtH4nE
Sparbier CE, et al. Nat Cell Biol. 2023 Feb;25(2):258-272. doi: 10.1038/s41556-022-01056-x. Epub 2023 Jan 12. PMID: 36635503; PMCID: PMC7614190.
Bhattacharya A, Commun Biol. 2023 Oct 11;6(1):1030. doi: 10.1038/s42003-023-05395-9. PMID: 37821650; PMCID: PMC10567710.

RNA PolII CTD Mouse monoclonal IgG1 (4H8) Cell Signaling 2629
https://www.cellsignal.com/products/primary-antibodies/rpb1-ctd-4h8-mouse-mab/2629?srsltid=AfmBOopmEKLzMjj34JLEQvykLb16Nz4yPNWII0N_i-zkOo5pMkylWmUG
Pang J, et al. Resveratrol intervention attenuates chylomicron secretion via repressing intestinal FXR-induced expression of scavenger receptor SR-B1. Nat Commun. 2023 May 9;14(1):2656. doi: 10.1038/s41467-023-38259-1. PMID: 37160898; PMCID: PMC10169763.
Zaurin R, et al. Nucleic Acids Res. 2021 Dec 16;49(22):12716-12731. doi: 10.1093/nar/gkab1125. PMID: 34850111; PMCID: PMC8682742.

RNA PolII phospho-Ser2 Rabbit polyclonal IgG Abcam ab5095
https://www.abcam.com/en-us/products/primary-antibodies/rna-polymerase-ii-ctd-repeat-ysptsps-phospho-s2-antibody-ab5095
Shin H, et al. Biochim Biophys Acta Gene Regul Mech. 2018 May;1861(5):481-496. doi: 10.1016/j.bbagrm.2018.03.002. Epub 2018 Mar 8. PMID: 29524612; PMCID: PMC6053077.
Baugh LR, et al. Science. 2009 Apr 3;324(5923):92-4. doi: 10.1126/science.1169628. Epub 2009 Feb 26. PMID: 19251593.

RNA PolII phosphor-Ser5 Rabbit polyclonal IgG Abcam ab5131
https://www.abcam.com/en-us/products/primary-antibodies/rna-polymerase-ii-ctd-repeat-ysptsps-phospho-s5-antibody-ab5131#
Fullwood MJ, et al. An oestrogen-receptor-alpha-bound human chromatin interactome. Nature. 2009 Nov 5;462(7269):58-64. doi: 10.1038/nature08497. PMID: 19890323; PMCID: PMC2774924.
Schones DE, et al. Cell. 2008 Mar 7;132(5):887-98. doi: 10.1016/j.cell.2008.02.022. PMID: 18329373; PMCID: PMC10894452.

Plants

Seed stocks

Report on the source of all seed stocks or other plant material used. If applicable, state the seed stock centre and catalogue number. If plant specimens were collected from the field, describe the collection location, date and sampling procedures.

Novel plant genotypes

Describe the methods by which all novel plant genotypes were produced. This includes those generated by transgenic approaches, gene editing, chemical/radiation-based mutagenesis and hybridization. For transgenic lines, describe the transformation method, the number of independent lines analyzed and the generation upon which experiments were performed. For gene-edited lines, describe the editor used, the endogenous sequence targeted for editing, the targeting guide RNA sequence (if applicable) and how the editor was applied.

Authentication

Describe any authentication procedures for each seed stock used or novel genotype generated. Describe any experiments used to assess the effect of a mutation and, where applicable, how potential secondary effects (e.g. second site T-DNA insertions, mosiacism, off-target gene editing) were examined.

ChIP-seq

Data deposition

☒ Confirm that both raw and final processed data have been deposited in a public database such as GEO.

☒ Confirm that you have deposited or provided access to graph files (e.g. BED files) for the called peaks.

Data access links
May remain private before publication.

ChIP and CUT&RUN data is available for reviewers at GEO SuperSeries GSE271090

Files in database submission

Scaled bedgraph files have been provided for all ChIP and CUT&RUN data, as well as MACS2 or SEACR defined peaks in the form of a bed file. Input or IgG control sample unscaled bedgraphs have been provided as well.

Genome browser session
(e.g. UCSC)

https://genome.ucsc.edu/s/maya.arce/hg38_cutnrun_IL2RA
note that not all files were loaded due to size restrictions, please see GEO data for full dataset

Methodology

Replicates

CUT&RUN was performed with 2 human donors (biological replicates) per KO, cell type, and stimulation condition for H3K27ac.
CUT&RUN was performed with 3 human donors (biological replicates) per KO, cell type, and stimulation condition for H34me1-3.
ChIPseq was performed with 2 human donors (biological replicates) per KO, cell type, and stimulation condition for each target.

Sequencing depth

All CUT&RUN samples were sequenced to a depth of 6-7 M PE reads per sample, with samples from the same donor and cell type run within the same batch . The human genome alignment rate was ~75-95% for each sample with 50-70% of reads aligning only once. The read length was 75 bp paired end for a total read length of 150 bp. An E.coli spike in was included in each sample at a rate of approximately 1% of reads.

ChIPseq samples were sequenced to a mean depth of 32 M PE reads per sample, with samples from the same donor and cell type run within the same batch . The alignment rate was between 87-100% for each sample with 32-100% of reads aligning only once. The read length was 50 bp paired end for a total read length of 100 bp. Mouse chromatin was spiked in at an estimated percentage of 2.5% of reads.

Antibodies

MED12 Rabbit polyclonal IgG Bethyl/Thermo A300-774A ChIPseq
CXXC1 Rabbit monoclonal IgG (D1R5R) Cell Signaling 40672S ChIPseq
NELF-A Mouse monoclonal IgG2bk (G-11) Santa Cruz sc-365004 ChIPseq
RNA PolII CTD Mouse monoclonal IgG1 (4H8) Cell Signaling 2629 ChIPseq
RNA PolII phospho-Ser2 Rabbit polyclonal IgG Abcam ab5095 ChIPseq
RNA PolII phosphor-Ser5 Rabbit polyclonal IgG Abcam ab5131 ChIPseq
H3K27ac Rabbit Monoclonal (2114-3E4) EpiCypher 13-0045 CUT&RUN
H3K4me1 Rabbit Monoclonal (2088-1F4) EpiCypher 13-0057 CUT&RUN
H3K4me2 Rabbit Monoclonal EpiCypher 13-0027 CUT&RUN

H3K4me3 Rabbit Monoclonal (2909-3D7) EpiCypher 13-0041 CUT&RUN
IgG Rabbit EpiCypher 13-0042 CUT&RUN

Peak calling parameters

CUT&RUN analysis
Pooled libraries were sequenced on a NextSeq 500 (H3K27ac) and NextSeq 2000 with 2x75 or 2x50 paired end reads, respectively. Bcl2fastq (v2.19) with the settings --minimum-trimmed-read-length 8 was used to generate fastqs. CUT&RUN data analysis was performed according Zheng et al. with the recommended settings unless otherwise specified below62. In brief, the fastqs were trimmed with cutadapt (v1.18). Bowtie2 (v2.2.5) was used to align the trimmed fastqs to GRCh38 using settings --local --very-sensitive --no-mixed --no-discordant --phred33 --dovetail -I 10 -X 700 -p 8 -q and E. coli (EMBL accession U00096.2) with settings --local --very-sensitive --no-overlap --no-dovetail --no-mixed --no-discordant --phred33 -I 10 -X 700 -p 8 -q. Bam files were generated with samtools(version 1.9) view -bS -F 0x04 and bam to bed conversion performed with bedtools (v2.30.0) bamtobed -bedpe. Bedfiles were filtered to include only paired reads of less than 1000 bp with the command awk '$1==$4 && $6-$2 < 1000 {print $0}' samplename.bed before generating bedgraph files using bedtools (version 2.30.0) genomecov -bg. Peak calling was performed using the bedgraph files as input with SEACR63 (v1.3). Each target bedgraph file was compared to the respective donor and KO condition IgG file to identify peaks above the background using the norm and stringent options for H3K27ac samples. Spike-in scaling was performed prior to methylation peak calling with SEACR using the IgG file as background and non and stringent options.

ChIP-seq analysis
Reads were trimmed to remove adapters and low-quality sequences and aligned to the hg38 and mm10 reference genome assemblies with bwa65 (v0.7.17-r1188) before filtering to remove duplicates and low-quality alignments including to problematic genomic regions66 using the nf-core/chipseq pipeline67 (v2.0.0, doi: 10.5281/zenodo.3240506) with default parameters. Normalization to mouse spike-in chromatin was performed by scaling counts to the quotient of the ratios of human:mouse ChIP reads and human:mouse input reads as described68. CXXC1 peaks for visualization were identified using bam files from all AAVS1 KO donors for MACS269 callpeak -q 0.05 with input samples used to define the background. High confidence MED12 peaks were identified using bam files from all AAVS1 KO donors for MACS2 callpeak -q 0.05 with MED12 KO samples used to define the background. Utilization of high confidence peaks generated from KO controls eliminated potential false positive signals from the ChIP samples, providing a more rigorous assessment of MED12 binding70,71. ChIPseq blacklist regions were removed from CXXC1 and MED12 peaks prior to analysis.

Data quality

SEACR, the gold standard peak caller for CUT&RUN data, was used to process CUT&RUN using the most stringent setting which included an IgG control for background thresholding. One IgG sample was generated for every donor, KO, cell type, and stimulation condition ample for the most accurate peak calling. MACS2, a standard peak calling program, was used for ChIPseq data with a sample matched input control file. For MED12 peak calling, a donor and condition matched MED12 KO sample was used as the background.

Software

CUT&RUN data analysis was performed according Zheng et al. (CUTTag_tutorial https://yezhengstat.github.io/CUTTag_tutorial) with the recommended settings unless otherwise specified. ChIPseq analysis was performed using the nf-core/chipseq pipeline (v2.0.0, doi: 10.5281/zenodo.3240506).

Flow Cytometry

Plots

Confirm that:

☒ The axis labels state the marker and fluorochrome used (e.g. CD4-FITC).

☒ The axis scales are clearly visible. Include numbers along axes only for bottom left plot of group (a 'group' is an analysis of identical markers).

☒ All plots are contour plots with outliers or pseudocolor plots.

☒ A numerical value for number of cells or percentage (with statistics) is provided.

Methodology

Sample preparation

CD4+ regulatory and effector T cells were isolated from fresh Peripheral Blood Leukopaks (STEMCELL Technologies, #70500) from healthy human donors, after institutional review board–approved informed written consent (STEMCELL Technologies). The contents of the Leukopaks were washed twice with a 1X volume of EasySep buffer (DPBS, 2% fetal Bovine Serum (FBS), 1mM pH 8.0 EDTA) using centrifugation. The washed cells were resuspended at 200E6 cells/mL in EasySep buffer and isolated with the EasySep™ Human CD4+CD127lowCD25+ Regulatory T Cell Isolation Kit (STEMCELL Technologies, #18063), according to the manufacturer's protocol. Following isolation with the kit, Tregs were stained Alexa Fluor® 647 anti-human CD25 Antibody (Biolegend, #302618), PE anti-Human CD127 (Beckon Dickinson, #557938), and Pacific Blue™ anti-human CD4 Antibody (Biolegend, #344620) and isolated using FACS to ensure a pure population without contaminating effector cells. After sorting pure CD4+CD127lowCD25+ Regulatory T Cells, the cells were seeded at 1x106 cells/mL in XVIVO-15 (Lonza, #02-053Q) supplemented with 55 uM 2-mercaptoethanol, 4 mM N-acetyl L-cysteine, and 200 U/mL IL-2 (Amerisource Bergen, #10101641). Teffs were seeded at 1x106 cells/mL in RPMI-1640 supplemented with 10% FCS, 2 mM L-Glutamine (Fisher Scientific #25030081), 10 mM HEPES (Sigma, #H0887-100ML), 1X MEM Non-essential Amino Acids (Fisher, #11140050), 1 mM Sodium Pyruvate (Fisher Scientific #11360070), 100 U/mL Penicillin-Streptomycin (Sigma, #P4333-100ML), and 50 U/mL IL-2 (Amerisource Bergen, #10101641). Both cell subsets were then stimulated with ImmunoCult™ Human CD3/CD28/CD2 T Cell Activator (STEMCELL Technologies, #10990) at 25 uL/mL for Tregs and 6.25 uL/mL for Teff. Following activation and electroporation, cells were split 1:2 every 48 hours to maintain an approximate density of 1x106 cells/mL and supplemented with respective doses of IL-2.

The Biolegend FoxP3 Fix/Perm kit (Biolegend, #421403) was used for staining according to the manufacturer protocol. Cells were washed in EasySep buffer prior to extracellular staining. Cells were stained with Alexa Fluor® 647 anti-human CD25 Antibody diluted 1:25 (Biolegend, #302618), Ghost Dye™ Red 780 diluted 1:1000 (Tonobo, #13-0865-T500) and BV711 anti-human CD4 diluted 1:50 (Biolegend, #344648) for 20 minutes at 4C and then washed once with EasySep buffer. After fixing and permeabilizing according to the kit, intracellular staining was performed with PE anti-mouse/human Helios Antibody (Biolegend #137216), KIRAVIA Blue 520™ anti-human CD152 (CTLA-4) Antibody (Biolegend #349938), Pacific Blue™ anti-human FOXP3 Antibody (Biolegend, #320116), and PE/Dazzle™ 594 anti-human/mouse Granzyme B Recombinant Antibody (Biolegend, #372216) diluted 1:50 in permeabilization buffer for 30 minutes at room temperature. Cells were subsequently washed in permeabilization buffer and resuspended in EasySep buffer before running on the ThermoFisher Attune NxT flow cytometer.

Instrument

Flow cytometry data was collected with the ThermoFisher Attune NxT flow cytometer (Cat #A29004). Cell sorting was performed on a BD FACS ARIA Fusion 1 (#656700).

Software

Cell sorter data collection was performed with BD FACSDiva. Attune Cytometric software was used for data collection with instrument compensation prior to sample collection. Analysis of flow data was performed in FlowJo (v10.8.1). Visualization was performed in R using ggplot2 (v3.4.1).

Analysis of flow data was performed in FlowJo (v10.8.1). Gating was performed to select for lymphocytes, singlets, live cells (Ghost Dye negative), and CD4+ cells in the specified order. This population was then used to calculate the median fluorescence intensity (MFI) for CD25 (IL2RA), CTLA-4, or Granzyme B. Visualization was performed in R using ggplot2 (v3.4.1).

Cell population abundance

For Treg sorting, purity was around 80-90% at the time of assay readout (generally 10-14 days post purity sort) as determined by FoxP3+Helios+ Treg populations after gating to select for lymphocytes, singlets, live cells (Ghost Dye negative), and CD4+ cells . During the sort, purity was routinely checked and accepted between 95-100% using surface markers CD4 +CD25hiCD127lo by running an aliquot of the collected sample back through the cell sorter. For screens, the same QC metrics were used during the sort where aliquots from IL2RA high and low bins were run back through the instrument to ensure distinct populations were collected.

Gating strategy

For marker quantification across conditions, gating was performed to select for lymphocytes, singlets, live cells (Ghost Dye negative), and CD4+ cells in the specified order. This population was then used to calculate the median fluorescence intensity (MFI) for CD25 or CTLA-4. Negative control samples were used to confirm gating strategies and gates were set using AAVS1 safe harbor control samples and applied to the rest of samples within the donor and cell type/stimulation condition. For apoptosis assays, cells were gated on lymphocytes and then a quadrant gate was used to distinguish Caspase-3/7+ and SYTOX- populations. Gate placement was determined using apoptosis negative control samples (no stimulation and AAVS1 safe harbor KO samples) and applied to all the samples from the donor. For all assays, gates were always applied consistently across KO conditions that were being compared. For example, all MED12 KO samples within the same donor, cell type, and stimulation condition were grouped with the AAVS1 KO samples that they would be compared to and the gates synced within groups.

☒ Tick this box to confirm that a figure exemplifying the gating strategy is provided in the Supplementary Information.
