# Supplementary material for: Central control of dynamic gene circuits governs T cell rest and activation
Source: Nature. 2024 Dec 11;637(8047):930–9. doi: 10.1038/s41586-024-08314-y (PMC11754113; doi:10.1038/s41586-024-08314-y)
Supplement: Supplementary file 3 — Supplementary Tables 1–12 [file 41586_2024_8314_MOESM3_ESM.zip › 2023-10-17819C-s3/SupplementaryTables-legend.docx]

**Central control of dynamic gene circuits governs T cell rest and activation**

Maya M. Arce^^[[1]](#footnote-1)^,^^^[[2]](#footnote-2)^,^^[[3]](#footnote-3)^, Jennifer M. Umhoefer^1,2,3^, Nadia Arang^[[4]](#footnote-4)^, Sivakanthan Kasinathan^1,^^[[5]](#footnote-5)^, Jacob W. Freimer^1,2,^[[6]](#footnote-6)^^, Zachary Steinhart^1,2^, Haolin Shen^3^, Minh T.N. Pham^[[7]](#footnote-7)^, Mineto Ota^1,2,6^, Anika Wadhera^1^, Rama Dajani^1^, Dmytro Dorovskyi^1,2^ , Yan Yi Chen^1,2^, Qi Liu^1,2^, Yuan Zhou^4,^^[[8]](#footnote-8)^, Danielle L. Swaney^4,8,^^[[9]](#footnote-9)^, Kirsten Obernier^4,8^, Brian R. Shy^1,^[[10]](#footnote-10)^^, Julia Carnevale^1,2,^[[11]](#footnote-11)^^^,^[[12]](#footnote-12)^^, Ansuman T. Satpathy^1,7,12^, Nevan J. Krogan^4,8,9,^^[[13]](#footnote-13)^ , Jonathan K. Pritchard^6,^^[[14]](#footnote-14)^, Alexander Marson^1,2,1^^1,12,^[[15]](#footnote-15)^,^[[16]](#footnote-16)^,^[[17]](#footnote-17)^^^,^[[18]](#footnote-18)^§^

**Supplementary Tables:**

*S1. S1_all_screens_results.xlsx*

IL2RA regulator screen sgrna and gene level results generated with MAGeCK. Each screen is indicated by the suffix (Resting_Treg, Resting_Teff, Stimulated_Teff). Results are representated as the IL2RA low bin/IL2RA high bin.

*S2. S2_pseudobulk_rnaseq_diff_expressed_regulators.xlsx*

Pseudo-bulked perturb-seq differential gene expression analysis output comparing each gene knock-down to non-targeting samples in the same cell type and stimulation condition. The data was generated using DESeq2 and includes only significantly differentially expressed genes (padj < 0.05) that were also IL2RA screen hits.

*S3. S3_bulk_RNASeq_Mediator_DESeq2_output_significant_differentially_expressed_genes.xlsx*

Bulk RNAseq differential expression analysis output comparing each gene knock-out to AAVS1 knock-out control samples in the same cell type and stimulation condition. The data was generated using DESeq2 and includes only significantly differentially expressed genes (padj < 0.05).

*S4. S4_MED12_IP-MS_SAINT_results.xlsx*

Peptides identified in MED12 immunoprecipitation mass spectrometry relative to IgG immunoprecipitation performed in the same condition. The Bait column indicates the purified protein and condition (R_MED12 = Resting, S_MED12 = Stimulated). The co-precipitated peptide is listed in the Prey column, followed by the corresponding gene name in the PreyGene column.

*S5. S5_pathway_analysis_results.xlsx*

Pathway enrichment analysis performed using MED12 knock-out differential expression bulk RNAseq data. PathfindR results generated for each cell type and stimulation condition using significant differentially expressed genes (padj < 0.05). Gene set enrichment analysis (GSEA) performed using all genes for each cell type and stimulation condition with msigdb gene sets.

*S6. S6 _sgrna_sequences.xlsx*

All sgRNA sequences and ids used in the described experiments. The modality column indicates use with cutting Cas9 (CRISPR KO) or dCas9 (CRISPRi).

S7. *S7_perturb_seq_activation_scoring_summary_table.xlsx*

Activation scores for each gene knock-down in perturb-CITE-seq experiment.

S8. *S8_stimulation_responsive_differential_expression.xlsx*

Bulk RNAseq differential expression analysis output comparing stimulated vs resting AAVS1 knock-out control Tregs and Teffs. The data was generated using DESeq2 and includes only significantly differentially expressed genes (padj < 0.05).

*S9. S9_antibody_table.xlsx*

Antibodies used in this study with application information.

*S10. S10_CUT&RUN_differential_regions.xlsx*

Differential H3K27ac and H3K4 methylation CUT&RUN analysis comparing each gene knock-out to AAVS1 knock-out control samples in the same cell type and stimulation condition, SEL120-34A treated samples to vehicle control treated samples, or control stimulated samples to resting samples (stimulation responsive sites). Includes only significantly different regions (padj < 0.05).

*S11. S11_MED12_high_confidence_peaks_ChIPseq.xlsx*

MED12 ChIPseq high confidence peaks identified in AAVS1 knock-out Teffs using MED12 knock-out samples to establish background signal. CXXC1 ChIPseq peaks identified in AAVS1 knock-out Teffs. The Gene.name column includes an annotation of the nearest transcription start site for the provided peak coordinates.

*S12. S12_Cytokine_luminex_results.xlsx*

Cytokine secretion results determined with Luminex. The concentration of the provided cytokine is listed for each knock-out and cell type condition. Treg and Teffs were processed in separate experiments.

1. Gladstone-UCSF Institute of Genomic Immunology, San Francisco, CA, USA. [↑](#footnote-ref-1)
2. Department of Medicine, University of California, San Francisco, CA, USA. [↑](#footnote-ref-2)
3. Biomedical Sciences graduate program, University of California, San Francisco, CA, USA [↑](#footnote-ref-3)
4. Quantitative Biosciences Institute (QBI), University of California, San Francisco, CA, USA [↑](#footnote-ref-4)
5. Division of Allergy, Immunology, and Rheumatology, Department of Pediatrics, Stanford University School of Medicine, Stanford, CA, USA [↑](#footnote-ref-5)
6. Department of Genetics, Stanford University, Stanford, CA, USA. [↑](#footnote-ref-6)
7. Department of Pathology, Stanford University School of Medicine, Stanford, CA USA [↑](#footnote-ref-7)
8. Gladstone Institute of Data Science and Biotechnology, San Francisco, CA, USA [↑](#footnote-ref-8)
9. Department of Cellular and Molecular Pharmacology, University of California, San Francisco, CA, USA [↑](#footnote-ref-9)
10. Department of Laboratory Medicine, University of California, San Francisco, CA, USA. [↑](#footnote-ref-10)
11. UCSF Helen Diller Family Comprehensive Cancer Center, University of California, San Francisco, CA, USA. [↑](#footnote-ref-11)
12. Parker Institute for Cancer Immunotherapy, San Francisco, CA, USA. [↑](#footnote-ref-12)
13. Department of Bioengineering and Therapeutic Sciences, University of California, San Francisco, CA, USA. [↑](#footnote-ref-13)
14. Department of Biology, Stanford University, Stanford, CA, USA. [↑](#footnote-ref-14)
15. Innovative Genomics Institute, University of California-Berkeley, Berkeley, CA, USA. [↑](#footnote-ref-15)
16. Department of Microbiology and Immunology, University of California, San Francisco, CA, USA. [↑](#footnote-ref-16)
17. 17 Institute for Human Genetics, University of California, San Francisco, CA, USA. [↑](#footnote-ref-17)
18. § e-mail: alex.marson@gladstone.ucsf.edu [↑](#footnote-ref-18)
